# Breaking into HIV-1’s Epigenetic Vault: Cure Strategies to Eliminate the Viral Reservoir

**DOI:** 10.3390/v18030354

**Published:** 2026-03-13

**Authors:** Joanna E. Jones, Chelsea E. Gunderson, Brian Wigdahl, Michael R. Nonnemacher

**Affiliations:** 1 Department of Microbiology and Immunology, Drexel University College of Medicine, Philadelphia, PA 19102, USA; jj993@drexel.edu (J.E.J.); ceg327@drexel.edu (C.E.G.); bw45@drexel.edu (B.W.); 2 Center for Molecular Virology and Gene Therapy, Institute for Molecular Medicine and Infectious Disease, Drexel University College of Medicine, Philadelphia, PA 19102, USA; 3 Sidney Kimmel Comprehensive Cancer Center, Thomas Jefferson University, Philadelphia, PA 19107, USA

**Keywords:** HIV-1, HIV-1 cure, chromatin, small molecules, epigenetic modifiers

## Abstract

Human immunodeficiency virus type 1 (HIV-1) is a retrovirus that integrates into the host cell’s DNA as a provirus. Transcription from the provirus is regulated in large part by cellular proteins and epigenetic factors. These may be repressive or permissive to productive infection. The host factors that regulate this balance are therefore attractive targets for HIV-1 therapeutics. Indeed, proviral chromatin is the focus of two of the current HIV-1 cure strategies. “Shock and Kill” uses latency reversal agents to open the provirus’s chromatin, promoting high levels of gene expression that induce the killing of infected cells. “Block and Lock” uses latency promoting agents to induce heterochromatin, blocking transcription and forcing HIV-1 into a state of deep latency. Here, the compounds investigated in both strategies are reviewed, including their chemical structures, mechanisms of action, and clinical results. Finally, the use of CRISPR-Cas therapeutics and the impact of chromatin architecture on its efficacy are discussed.

## 1. Introduction

Since the first diagnosed cases of what would become known as Acquired Immunodeficiency Syndrome (AIDS) in 1981, Human Immunodeficiency Virus type 1 (HIV-1) infection has resulted in over 40 million deaths globally [1,2]. However, with increased awareness of HIV-1 testing, prevention, and antiretroviral therapy (ART), the number of deaths due to HIV-1/AIDS-related causes has decreased significantly since the peak of the pandemic, and the life expectancy of people living with HIV-1 (PWH) is now comparable to that of the general population. However, with nearly 40 million people living with HIV-1 and an estimated 630,000 deaths in 2023 [2,3], HIV-1 remains a global health concern.

Understanding the infection and replication cycle of HIV-1 has been critical to the development of effective ART. HIV-1 enters the host cell by first binding to CD4+ as a primary receptor, then CCR5 or CXCR4 as a coreceptor; as such, HIV-1’s tropism is restricted to cells of the immune system [4], such as CD4 T cells and macrophages. Fusion of the viral envelope with the plasma membrane deposits the viral core into the cytoplasm (reviewed in [5]). The viral core contains two copies of HIV-1’s RNA genome (genRNA), the viral enzymes reverse transcriptase (RT) and integrase (IN), and other enzymes and proteins captured during the capsid assembly process (reviewed in [6]). RT converts the genRNA to linear, double-stranded DNA (gDNA) as the core is trafficked to the nuclear membrane by the cell’s own actin cytoskeleton; after translocation through the nuclear pore, the core uncoats to release the gDNA genome, RT, and IN into the nucleoplasm (reviewed in [7,8]). IN, along with certain cellular enzymes, catalyzes the integration of the gDNA into the host’s genome as a provirus [9,10,11]. Notably, RT is highly error-prone, leading to the evolution of diverse quasispecies in PWH [12,13].

As a provirus, HIV-1 uses the host cell’s transcriptional and translational machinery to make viral mRNAs, genRNAs, structural proteins, and viral enzymes. In addition, certain viral proteins promote gene expression from the provirus; for example, the viral Trans-Activator of Transcription (Tat) recruits the cellular Positive Transcription Elongation Factor b (P-TEFb) to the core promoter, enabling phosphorylation of RNA polymerase II (RNAPII) and therefore processive transcription from the provirus—a process collectively referred to as transactivation (reviewed in [14]). During productive and acute infection, capsid proteins assemble at the plasma membrane, and the viral protease (PR) cleaves the Gag-Pol polyprotein to release the mature virion into the extracellular space (reviewed in [15]).

However, after the acute infection is cleared, some cells escape the immune response and continue to produce viral particles, while others transition to a state of quiescence; as transcription factors become less plentiful and epigenetic changes drive other transcriptionally repressive changes in the nucleus, the provirus in these cells also enters a period of latency, characterized by low levels of viral gene expression and virion production (reviewed in [16,17]). While the latent period may last a decade or more, in the absence of therapeutic intervention, progressive viral expansion leads to the death of host cells, thereby crippling the immune system, resulting in progression to AIDS [18].

The first FDA-approved antiretroviral drug, azidothymidine (AZT), became available in 1987. As a nucleoside analog of thymidine, AZT may be incorporated into viral gDNA during reverse transcription, resulting in premature termination. AZT was highly effective at controlling disease progression, but concerns about drug resistance due to the monotherapy began to arise. Indeed, in 1995, it was found that the introduction of dideoxycytidine (ddC), another nucleoside reverse transcriptase inhibitor (NRTI), alongside AZT was more effective than AZT alone. Just one year later, in 1996, the PR inhibitor saquinavir was introduced into ART regimens, and use of the three drugs together became known as highly active antiretroviral therapy (HAART) or combined antiretroviral therapy (cART) [19,20].

Since the introduction of cART, many other ART drugs have been developed to target other stages of the HIV-1 replication cycle. These include entry and fusion inhibitors, integrase inhibitors, and non-nucleoside reverse transcriptase inhibitors (NNRTI) [19]. Use of multiple ART drugs remains the standard of care for PWH to this day. Now, ART is also available in the form of pre- and post-exposure prophylaxis (PrEP and PEP, respectively) for people without HIV-1 (PWoH) [20], and PWH may also opt for long-acting ART such as lenacapavir, a novel capsid inhibitor [21].

Importantly, however, ART is not curative; it is unable to target the integrated provirus. Thus, simple clonal expansion of infected cells maintains a reservoir of provirus that may continue to produce viral proteins. Additionally, should a person taking ART choose not to adhere to the drug regimen, viral rebound occurs within weeks. This may result in disease progression in the absence of medical intervention [22]. Therefore, a true cure for HIV-1 must target and clear the reservoir of infected cells.

In February of 2007, Timothy Ray Brown, who would become known as the Berlin Patient, became the first person to ever be cured of HIV-1. He had been diagnosed with acute myeloid leukemia in 2006 and had been living with HIV-1 since 1995. In an effort to treat his leukemia, Brown’s hematologist, Dr. Gero Hutter, screened for bone marrow donors who were not only compatible for a transplant but also had the CCR5Δ32 mutation. CCR5Δ32 results in a truncated, nonfunctional CCR5 receptor and confers resistance to HIV-1 infection and disease progression [23,24]. Indeed, after two bone marrow transplants, Brown remained free of HIV-1 until his death in September of 2020 [25].

Since Brown’s initial transplant, several other leukemia and lymphoma patients have additionally been cured of HIV-1 in a similar manner [26]. With the advent of effective gene-editing techniques, ex vivo gene editing and autologous stem cell transplant have been proposed as potential cure strategies for HIV-1 [27,28,29,30,31,32]. However, this approach has several notable limitations. First, it is highly invasive, requiring myelosuppression or myeloablation to avoid rebound after transplant (reviewed in [23]). Second, recipients are at risk of other opportunistic infections, cardiac events, and certain cancers [33,34]. Third, stem cell transplants are costly with regard to accessibility, finances, treatment duration, and recovery time [35,36]. Finally, autologous transplant availability is limited in sub-Saharan Africa [37,38], where HIV-1 is most prevalent globally. Taken together, there is a necessity for cure strategies that are safe, effective, and accessible for PWH.

Because HIV-1 integrates into the host genome, targeting the provirus directly poses a challenge to therapeutic development. However, in the decades since the first cases of AIDS were identified, the field of HIV-1 latency research has expanded, and it has become well-established that host- and virus-driven epigenetic mechanisms are key in controlling the activation state of the provirus. Targeting these processes therapeutically provides a promising, novel strategy to modulate HIV-1 latency and reactivation. It has culminated in the development of two chromatin-centric HIV-1 cure strategies: “Shock and Kill” and “Block and Lock.” In this work, we review the literature on a select group of small molecules and other compounds investigated for use in these strategies.

## 2. A Primer on Chromatin and Epigenetic Regulation of HIV-1 Gene Expression

Epigenetic regulation of transcription from the integrated provirus is highly complex and involves a myriad of cellular enzymes, adaptor proteins, transcription factors (TFs), and the viral protein Tat. To provide context and terminology for HIV-1 cure strategies targeting these processes, we will briefly and non-exhaustively describe key epigenetic factors that positively and negatively regulate proviral gene expression.

**Figure 1:**
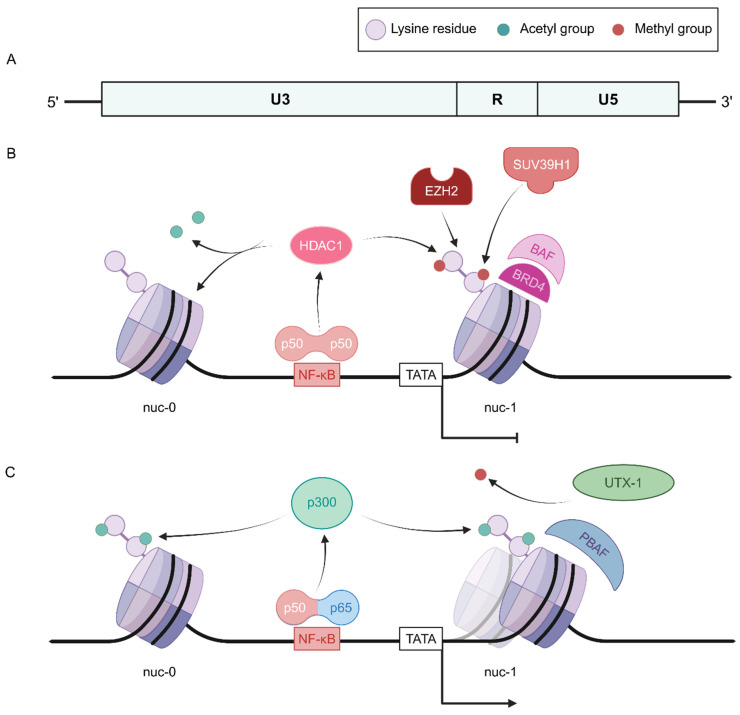
Nucleosomes on the 5′ LTR regulate transcription. (**A**) Simplified diagram of the LTR featuring regions U3, R, and U5. (**B**) The 5′ LTR of latent proviruses primarily exists in a heterochromatic state. Nuc-0 and nuc-1 are precisely positioned in U3 and R-U5, respectively, repressing transcription by blocking RNAPII access. Nuc-1 is maintained in its position by BRD4 and the BAF complex. NF-κB p50 is the predominant subunit in the nucleus during T cell quiescence; hence, it constitutively occupies the NF-κB binding sites of latent proviruses. p50 and other factors recruit HDACs, which remove acetyl groups from nuc-0 and nuc-1 to promote tighter interactions with nucleosomal DNA. Methyltransferases such as EZH2 and SUV39H1 catalyze the addition of methyl groups to certain residues, which suppresses transcription through steric interference or by recruiting other factors to compact chromatin. (**C**) During latency reversal, the 5′ LTR takes on a euchromatic state. The PBAF complex remodels nuc-1 to a position slightly downstream of the TSS to enable access by RNAPII. NF-κB p65 dimers replace p50 and recruit acetyltransferases such as p300, which promotes euchromatin formation by neutralizing electrostatic interactions between nucleosomal histones and DNA. Demethylases such as UTX-1 also remove interfering methyl groups, promoting p300 activity and allowing other factors to interact.

After the linear, double-stranded DNA genome of the virus is delivered to the nucleus, it is loaded with histone proteins, forming nucleosomes at the 5′ LTR and beyond (Figure 1) [39,40,41]. Nucleosomes are the most basic subunit of chromatin and are comprised of a linker histone H1 and two copies of each core subunit: H2A, H2B, H3, and H4. These proteins are rich in lysine and arginine residues, conferring an overall positive charge to the histone and enabling its interaction with the negatively charged nucleosomal DNA [42,43]. Additionally, the N-terminus of histone proteins protrudes from the DNA-bound nucleosome structure, exposing them to enzymes that catalyze the addition or removal (often referred to as “writing” or “erasing,” respectively) of post-translational modifications on key residues. These modifications alter the affinity of the histone for nucleosomal DNA, and, therefore, the accessibility of the bound region to TFs and RNA polymerase II (RNAPII). Some modifications additionally recruit transcriptional activators and repressors to the site, and others may work in tandem to achieve other complex effects (for a detailed review on this topic, see reference [44]). To this end, the nucleosome may also be physically repositioned over a region of DNA by ATP-dependent chromatin-modifying complexes (CMCs), such as the SWItch/Sucrose Non-fermentable (SWI/SNF) family or the Facilitates Chromatin Transcription (FACT) complex. This in turn exposes or hides TF binding sites, transcription start sites (TSSs), and TATA boxes (for a detailed review on this topic, see reference [45]).

Indeed, along with LEDGF/p75, interactions between the viral integrase and CMCs such as SWI/SNF family members enable integration into the chromatinized host DNA [9,10]. As the provirus transitions to latency, the SWI/SNF family member BRG1- or BRM-associated Factor (BAF) is recruited to the 5′ LTR by Bromodomain-containing Protein 4 (BRD4) and repositions the nucleosomes at the 5′ LTR to specific, highly conserved locations: upstream of the U3 region (nuc-0) and the R-U5 region (nuc-1) (Figure 1B) [46,47,48]. In particular, nuc-1 has long been observed to repress transcription from the provirus because it impedes transcriptional machinery by blocking the TSS, TAR region, and other TF binding sites [46,49,50].

Previously, we have reviewed the histone modifications known to promote proviral latency and the host enzymes that read, write, and erase them (see reference [16]). The effects of histone deacetylation are particularly well-characterized, and indeed a number of host factors have been associated with the recruitment of histone deacetylase complexes (HDACs) to the provirus; these include, but are not limited to, Ying Yang 1 (YY1) [51], C-promoter Binding Factor 1 (CBF-1) [52], B cell lymphoma/leukemia 11B (BCL11B or CTIP2) [53], and NF-κB p50 homodimers [54]. The HDACs recruited to the integrated provirus, particularly class I HDACs like HDAC1 and HDAC2, remove acetyl groups from nuc-0 and nuc-1, which compacts chromatin and represses proviral gene expression as the histone proteins regain affinity for the nucleosomal DNA, thus decreasing accessibility to the TSS and TAR by transcriptional machinery (Figure 1B) (for a detailed review on HDAC structure and mechanisms of action, see reference [55]).

Some residues, such as lysine-9 and lysine-27 on histone H3 (H3K9 and H3K27, respectively), may be subject to methylation as well as acetylation/deacetylation—although these modifications are mutually exclusive. Methylation of H3K9 and H3K27 is considered to be repressive to transcription, as this modification may sterically hinder TFs from interacting with the local DNA or recruit other factors to promote the formation of heterochromatin (reviewed in [44,56,57]). Indeed, Suppressor of Variegation 3-9 Homolog 1 (SUV39H1) and G9a, and Polycomb Repressive Complex 2 (PRC2, the catalytic unit of which is EZH2), which catalyze H3K9 and H3K27 methylation, respectively, are both enriched at the latent provirus [58,59,60]. In accordance with this, H3K9me3 and H3K27me3 accumulate on latent proviruses as the host cell—particularly host T cells—transitions to quiescence [61].

DNA methylation is also thought to play a role in the maintenance of HIV-1 latency, as multiple studies have shown that modulation of methylated CpG sites within the 5′ LTR results in proviral reactivation [62,63,64,65,66,67,68,69,70,71,72,73,74,75]. In eukaryotes, DNA methyltransferases (DNMTs) catalyze the addition of a methyl group onto the fifth carbon of a cytosine (5mc) within a CpG dinucleotide [76,77,78,79]. Sites consisting of higher numbers of CpG dinucleotides, termed CpG islands, are often found within promoter regions and are typically hypomethylated, allowing for TFs and other DNA-binding proteins to drive transcription. Hypermethylation of these sites results in transcriptional silencing through inhibited TF binding, recruitment of methyl-binding proteins that promote repressive histone modifications, and heterochromatin formation (reviewed in [80,81,82]). Several studies of in vitro models of HIV-1 latency have demonstrated hypermethylation of CpG islands encompassing TF binding sites within the 5′ LTR, and reactivation from latency upon treatment with DNA methyltransferase inhibitors [62,63,65,66,68,69,70,83,84,85]. Moreover, other studies have shown that treatment with a potent activator such as TNF-α results in demethylation of CpG islands within the 5′ LTR [69,86,87].

While much of the literature points to the involvement of methylation for maintaining latency in vitro, one study reported no correlation of CpG methylation with latency in T cell lines and primary CD4+ T cells infected ex vivo [88]. Evidence for the contribution of proviral methylation in latency maintenance in vivo is also varied. Blazkova and colleagues assessed methylation patterns of the 5′ LTR in latently infected Jurkat T cells in comparison with resting CD4+ T cells from a small cohort of virus-suppressed PWH (VS-PWH), observing a negative correlation between methylation state and reactivation in both in vitro and ex vivo studies [83]. In aviremic CD4+ T cells, proviruses were hypermethylated and resistant to reactivation, as opposed to the hypomethylated 5′ LTRs found in viremic samples. They also reported that the inhibitory effects of hypermethylation could be overcome by treatment with potent activators, including HDAC inhibitors, PKC agonists, and TNF-α in combination with a DNMT inhibitor [83]. However, this group performed a subsequent study using a larger cohort of aviremic PWH on long-term ART (LT-ART), and found that hypermethylation of the 5′ LTR was rare in such cases [89]. Another study found a negative correlation between LTR methylation and proviral transcription in HIV-1-infected sperm and subsequently derived 2-cell embryos [90]. Interestingly, Weber and colleagues reported low levels of CpG methylation in the LTR along with *gag*, *env*, *nef*, *rev* and *tat* coding sequences in a diverse cohort of PWH, including elite controllers, long-term non-progressors, and individuals with progressive infection, with the exception of one non-progressor who was found to have a high degree of fluctuation in CpG methylation over more than a decade [91].

Other studies have likewise pointed to a temporal aspect in the contribution of CpG methylation to latency. Trejbalova et al. found that CpG methylation of the 5′ LTR accumulates over time after successive stimulations with potent activators in vitro, resulting in a stable methylated state of the 5′ LTR in this model [72]. This was similarly reflected in resting CD4+ T cells taken from PWH on ART, as time on ART was positively correlated with levels of LTR methylation [72]. These results are in agreement with another study that demonstrated a positive correlation between 5′ LTR CpG methylation and time of infection in elite controllers and long-term non-progressors [92].

While most of the literature has focused on CpG methylation in the 5′ LTR, CpG islands are also found within viral genes and intragenic regions of the provirus [71,91,93]. A more recent study utilizing next-generation bisulfite sequencing in a cohort of PWH consisting of long-term non-progressors, recent seroconverters, and individuals on early and late cART demonstrated higher levels of intragenic proviral CpG methylation relative to levels on the 5′ LTR across non-progressors and individuals on cART [73]. Recent seroconverters, in contrast, had higher levels of CpG methylation in the 5′ LTR relative to intragenic regions [73]. Together, these studies point to a nuanced contribution of CpG methylation across the proviral genome in the regulation of latency over time.

In addition to these localized structures, the higher-order, 3D chromatin environment of HIV-1’s integration site plays a significant role in proviral latency. We have previously reviewed how 3D nuclear architecture influences proviral integration and latency in model systems and in PWH (see reference [16]). Perhaps paradoxically, transcriptionally active regions are regarded as a preferential substrate for integrase [94,95,96,97,98], but latent proviruses are often found in heterochromatin [94,99]. A recent study by Kizito et al. revealed that entry into quiescence drives significant chromatin compaction in CD4+ T cells, accompanied by the physical repositioning of the integrated provirus to transcriptionally restricted regions of the nucleus [100]. Likewise, selective pressure from LT-ART results in a greater number of proviruses in heterochromatic regions in PWH [101,102].

When latency is reversed, such as through stimulation by chemicals such as mitogens or TCR engagement (reviewed in [103,104,105]), the chromatin environment of the integrated provirus undergoes significant changes. The integration site is repositioned to the transcriptionally active A compartment in the nucleus [100], and transcriptional activators such as NF-κB p65 and Sp1 replace their repressive counterparts at the 5′ LTR [106,107]. This is additionally associated with enrichment of histone acetyltransferases (HATs) like p300/CREB-binding protein (CBP) [108,109,110] and the H3K27 demethylase UTX-1 [111], which work together to “loosen” the interaction between DNA and histone by neutralizing the charge of the histone core. The BAF complex is also displaced by Polybromo-associated BAF (PBAF), which moves nuc-1 slightly downstream of its canonical position and unblocks the TSS (Figure 1C) [112,113,114]. Interestingly, these changes are not associated with global chromatin opening in T cells, but microglia—and possibly macrophages—undergo significant chromosomal remodeling upon HIV-1 reactivation (reviewed in [16]).

Altogether, these local (2D) and global (3D) alterations enable access to the provirus by transcriptional machinery, thus promoting high levels of HIV-1 gene expression and replication of the full-length RNA genome. To this end, it has long been observed that small molecules, such as HDAC inhibitors and PKC agonists, trigger remodeling of nuc-1. This results in increased accessibility to the TSS and TAR, which favors processive transcription from the provirus [46,49,50]. As we have discussed previously, the multitude of epigenetic processes that regulate proviral latency provides a diverse set of therapeutic targets to be investigated towards the development of an HIV-1 cure.

## 3. Shock and Kill

The strategy of “Shock and Kill” (also known as “Kick and Kill”) is a two-step process. In the first step, Shock, HIV-1 latency is deliberately reversed using chemical agents, resulting in increased proviral gene expression and virion production, similar to what is observed during the initial acute infection. Immune recognition of viral proteins and products of RT activity, such as by Toll-like receptors, induces production of interferons and other cytokines that recruit and activate antigen-presenting cells, natural killer (NK) cells, and HIV-1-specific CD8+ T cells (reviewed in [115,116]). In the second step, “Kill,” the cytotoxic activity of NK and CD8+ T cells targets infected cells for killing and clearance; likewise, productive infection itself may result in cytolysis or apoptosis of the infected cell (reviewed in [117]).

Many of the cellular factors that maintain HIV-1 latency have also been implicated in other disease states, such as different cancers and congenital disorders, and have been therapeutically targeted by small molecules and other compounds. Many of these molecules have been demonstrated to facilitate the formation of euchromatin, either by directly loosening the nucleosome structure or inducing pathways that induce pro-transcriptional CMCs and histone-modifying enzymes. As such, these effects warrant the question: can these molecules also “shock” HIV-1 out of latency?

Indeed, over the past two decades or so, several anticancer compounds, natural products, and other small molecules have been investigated for their ability to reverse HIV-1 latency; collectively, they are known as latency reversal agents (LRAs). In this section, we review the literature focusing on a selection of LRAs with historical significance, clinical and virological relevance, and/or unique targets. Importantly, this is by no means an exhaustive list of all LRAs under investigation as of the completion of this work, and the exclusion of any particular LRA does not reflect its scientific or therapeutic potential.

### 3.1. HDAC Inhibitors

As we have discussed previously, the latent provirus is enriched with class I HDACs, which maintain nuc-0 and nuc-1 in a hypoacetylated, condensed state (Figure 1A). Class I HDACs require a zinc ion and water to coordinate a nucleophilic attack on the substrate histone acetyl-lysine’s carbonyl, which cleaves the acetyl group and releases the now-unmodified histone lysine (reviewed in [55,118]). Inhibitors of class I HDACs (collectively, HDACis) typically bind to the zinc ion, blocking the active site from accepting acetyl-lysine substrates. Other elements of HDACi structures additionally secure the molecule in the active site, providing a highly stable and long-lasting effect (reviewed in [55]).

It has long been observed that small-molecule HDACis reverse HIV-1 latency. Early studies of HIV-1 latency regulation by chromatin showed that the HDACis trichostatin A (TSA) and trapoxin (TPX) not only induced p24 and viral RNA production, but also appeared to significantly increase the sensitivity of the R region to DNase and restriction enzyme digestion, suggesting that nuc-1 was moved from its canonical position [49]. Further studies confirmed that TSA treatment resulted in HDAC1 eviction from nuc-1 and a concomitant increase in H4 acetylation [109,119,120].

Since these early investigations, many other small-molecule HDAC inhibitors have been discovered or developed to treat other diseases, particularly different cancers, as we discussed previously. Here, we review the literature supporting a selection of HDACis known to reverse HIV-1 latency (summarized in Table 1 below).

#### 3.1.1. Valproic Acid

Valproic acid (VPA) is a simple short-chain fatty acid known to inhibit class I and IIa HDACs, and is therefore considered a pan-HDACi (Table 1) [131]. Within the active site of class I HDACs, it binds to the zinc ion and is stabilized by interactions between the carboxylic group and amino acids in the catalytic tunnel [132]. As a drug, VPA is classed as an anticonvulsant and is used in the treatment of seizures, bipolar mania, and migraine headaches [133].

VPA was initially recommended to prevent seizures in PWH, and was even found to increase the concentration of AZT in plasma (reviewed in [134]). However, previous in vitro studies raised concerns about the safety of this treatment regimen. Indeed, one study demonstrated that the lymphocytic CEM-SS [135] and monocytic U1 cell lines treated with VPA experienced a dose-dependent increase in reverse transcriptase activity and p24 production; Jurkat T cells expressing β-galactosidase under control of the LTR (LTR-β-gal) had a similar increase in fluorescence as observed via MUG assay [121]. This was further corroborated by Moog et al., who observed the same effect in HIV-1-infected PBMCs [136].

It was initially postulated that the mechanism through which VPA stimulates HIV-1 gene expression is by decreasing glutathione (GSH) levels, as this effect was seen in both Jurkat and U937 cells; this hypothesis was in line with previous research demonstrating that reduction of GSH by treatment with diamide, a thiol-oxidizing reagent, sensitizes HIV-1 to activation by TNF-α stimulation, whereas restoration of GSH had the opposite effect [137]. However, Moog et al. later determined that GSH reduction by VPA was independent of HIV-1 reactivation, as VPA had different effects on GSH levels depending on the cell type used [136].

These initial investigations into VPA’s effects on HIV-1 activation were conducted prior to its identification as an HDACi. However, in 2001, a landmark study by Phiel et al. confirmed that VPA potently inhibited HDAC activity in cell-free extracts and Neuro2A cells alike, leading to hyperacetylation of histone H4 [138]. Taken together with previous findings, as well as the understanding that the HDACi TSA could reverse HIV-1 latency, this led to the hypothesis that VPA induces HIV-1 activation not through GSH depletion, but by facilitating acetylation of nuc-0 and nuc-1 [122].

Indeed, chromatin immunoprecipitation of VPA-treated J89 cells, which contain a latent provirus expressing GFP under control of the 5′ LTR [139], showed that acetylation of histones H3 and H4 was significantly increased at the nuc-1 region after just a few hours. Unsurprisingly, this was also associated with decreased occupancy of HDAC1 at nuc-1 [123]. In agreement with previous findings, VPA treatment also led to viral outgrowth in PBMCs isolated from VS-PWH [122,123] and GFP expression in J89 cells [123].

In an in vivo pilot study by Lehrman et al., it was demonstrated that VPA could also induce HIV-1 gene expression in VS-PWH at standard, clinically relevant doses, along with an intensified ART regimen that included the fusion inhibitor enfuvirtide. At the end of the study, all but one participant had a significant reduction in the frequency of HIV-1 in the resting CD4+ T cell population (i.e., resting CD4+ T cell infection, or RCI) [140]. However, further clinical trials indicated that the standard, rather than intensified, ART regimen is insufficient to have this effect in some PWH [141], and any reduction in RCI following VPA and intensified ART was not sustained over a period of 48–96 weeks [142]. Likewise, there was no observed increase in the HIV-1-specific T cell response following VPA and ART treatment [141].

It is possible that VPA is simply not potent enough to achieve the desired effect of “Shock and Kill.” Several in vitro studies have demonstrated that it is only effective in vitro at millimolar concentrations, whereas TSA and suberoylanilide hydroxamic acid (discussed in the following section) can inhibit HDACs at nanomolar concentrations [122,123,138]. Therefore, a stronger “shock” may be necessary to induce high enough levels of HIV-1 gene expression and virion production to elicit an immune response.

#### 3.1.2. Suberoylanilide Hydroxamic Acid

Suberoylanilide hydroxamic acid (SAHA) is a hydroxamic acid that inhibits class I HDACs, with some activity against class IIb and IV HDACs (Table 1) [123,143]. It inhibits HDACs by replacing water with its own hydroxyl group in the active site, and is “locked” into the catalytic tunnel by its aromatic group, enabling sustained inhibitory activity (reviewed in [55]). SAHA is also referred to as vorinostat and is commercially available under the brand name Zolinza as a treatment for cutaneous T cell lymphoma (CTCL) [144].

In an early study of SAHA’s potential to reverse HIV-1 latency compared to VPA, it was found that SAHA was much more potent and could activate proviral gene expression at nanomolar concentrations in J89 cells, whereas VPA required millimolar concentrations, as we discussed previously. It also induced acetylation of H3 at the nuc-1 region, and unsurprisingly caused HDAC1 to be removed from the proviral promoter [123]; likewise, another study demonstrated that treatment induced acetylation of histone H4 and remodeling of nuc-1 in Sup-T1 T cells [124]. In agreement with these findings, SAHA could induce viral outgrowth in CD4+ T cells from well-suppressed participants with HIV-1. Notably, SAHA has also been demonstrated to induce proviral gene expression in U1 cells, suggesting its use as an LRA in multiple cell types that comprise the HIV-1 reservoir.

Another possible mechanism through which SAHA may reverse latency is its activation of the PI3K/Akt pathway by promoting phosphorylation of protein kinase B (Akt), or inhibiting HDAC1 to promote acetylation of p65 [145]. Indeed, Contreras et al. observed that SAHA treatment induced activation of Akt in all T cell populations tested, including memory T cells, without inducing activation of the cells themselves. In accordance with this finding, SAHA treatment caused Cdk9 to be released from the large inactive complex, and subsequently promoted recruitment of P-TEFb to the proviral promoter [125].

Importantly, SAHA has also been demonstrated to increase naïve cells’ susceptibility to HIV-1 infection. In primary CD4+ T cells (pCD4s), pre-treatment with SAHA increased the frequency of infection events. Additionally, proviruses in these cells had enhanced LTR-driven transcription [127], indicating that SAHA not only reverses latency, but also prevents latency establishment after integration. Interestingly, viral entry was unaffected by treatment, but the cells were less sensitive to the RT inhibitor efavirenz [127], indicating that SAHA’s ability to promote infection lies downstream of reverse transcription.

An early, short-term clinical trial demonstrated that treatment could upregulate HIV-1 RNA expression within infected cells, which was accompanied by an expected increase in global histone H3 acetylation. Notably, HIV-1 RNA levels in plasma remained low, and SAHA did not induce viremia [146]. A subsequent clinical trial revealed that SAHA treatment failed to induce a sustained HIV-1-specific immune response and was unable to significantly affect the size of the reservoir as measured by integrated HIV-1 DNA [147]; this was corroborated in an additional study wherein some participants were also treated with hydroxychloroquine and maraviroc [148].

Another notable finding that came out of these trials is that SAHA treatment ultimately perturbs host chromatin modifications and gene expression [146,147,149], which may impact the immune system’s ability to recognize and mount a response against HIV-1 infection. Indeed, a study by Jones et al. showed that SAHA, as well as other clinically relevant HDACis, impair both interferon production and proliferation in HIV-1-specific CD8+ T cells, even after pre-activation [150]. Therefore, effective use of SAHA as an in vivo LRA may require additional compounds to promote immune-mediated killing of infected cells.

#### 3.1.3. Panobinostat

Panobinostat, also known as LBH589, is a small-molecule pan-HDACi with efficacy against class I, II, and IV HDACs (Table 1) (reviewed in [151]). Like SAHA and TSA, panobinostat is a hydroxamic acid derivative and therefore similarly inhibits HDACs through binding of the zinc ion within the catalytic domain (reviewed in [55,152,153]). As of 2015, it is FDA-approved for the treatment of multiple myeloma and commercially available under the brand name Farydak (reviewed in [154]).

When compared to SAHA and other hydroxamate HDACis, panobinostat was able to achieve virus production at concentrations much lower than the current therapeutic dose in ACH2 and U1 cells, and was found to be much more potent than SAHA [126]. In addition, panobinostat could stimulate transcription from diverse LTRs isolated from PWH [128]. These results were corroborated in PBMCs isolated from PWH, and treatment unsurprisingly resulted in hyperacetylation of histone H3 at nanomolar concentrations [129]. In contrast to SAHA and VPA, panobinostat could also induce the activation marker CD69 in CD4+ T cells, including memory T cells, ex vivo [126,155]. Interestingly, treatment reduced CCR5 expression on monocytes, but not T cells [126], indicating a potential protective role as well as latency reversal ability.

In agreement with these findings, administration of panobinostat to BLT humanized mice induced acetylation of histone H4 in multiple tissue types. Interestingly, treatment did not increase the amount of cell-associated HIV-1 RNA or DNA [129], which is in contrast to previous in vitro and ex vivo results. In a phase 1/2 clinical trial, however, panobinostat treatment did significantly increase cell-associated HIV-1 RNA levels, but ultimately did not affect the size of the reservoir [156]. Additional studies showed that panobinostat did not affect the HIV-1-specific CD8+ T cell or B cell response [157], and actually seemed to decrease inflammatory markers and increase T regulatory cell (Treg) levels [155,158]. Notably, Olesen et al. found that any reduction in HIV-1 DNA was correlated with innate immune function, particularly NK and dendritic cell activity [157]. Taken together, enhancing the pro-inflammatory and cytotoxic capabilities of innate immune cells may be an effective kill strategy.

#### 3.1.4. Romidepsin

Romidepsin, also known as depsipeptide and FK228, is a bicyclic depsipeptide isolated from the fermentation broth of *Chromobacterium violaceum*, a common environmental bacillus species [159,160,161]. Compared with other HDACis, romidepsin is unique in that it is a prodrug. Within the cell, its disulfide bond is reduced to a dithiol, generating the active form (reviewed in [161]). It is highly specific for class I HDACs (Table 1), and is predicted to bind to the catalytic zinc ion by one of its thiol groups (reviewed in [55]). It was first identified in 1994 by Ueda et al. and was found to be highly cytotoxic in carcinoma lines, but tolerated in healthy cells [159]. Romidepsin was later found to increase acetylation of all histone cores both in vitro and in vivo [162,163]. Like SAHA, romidepsin has been FDA-approved for the treatment of CTCL and is commercially available under the name Istodax (reviewed in [161]).

As a potent and specific HDACi, romidepsin is attractive as a potential LRA. Indeed, in ex vivo-infected pCD4s, romidepsin could induce maximal LTR-driven transcription at lower concentrations than SAHA, panobinostat, and other HDACis, and could induce and sustain p24 expression at nanomolar concentrations. These results were corroborated in CD4+ T cells isolated from aviremic participants, in which HIV-1 reactivation and HDAC inhibition were both sustained for over 24 h after a short treatment period. Notably, romidepsin seemed to selectively activate T cells, but not B cells, without significant induction of pro-inflammatory cytokines [130].

In an early clinical trial on romidepsin’s effects on HIV-1 reactivation in vivo, periodic romidepsin dosing resulted in an expected cyclical increase in histone H3 acetylation, which correlated with increases in cell-associated HIV-1 RNA levels. Romidepsin also stimulated CD69 expression in CD4+ and CD8+ T cells alike, in agreement with previous ex vivo findings [164]. However, in a recent phase 1/2 clinical trial, romidepsin failed to affect both cell-associated and plasma HIV-1 RNA levels despite inducing H3K9 acetylation and P-TEFb activation, and actually seemed to decrease the number of circulating CD4+ T cells in participants [165]. In both trials, romidepsin alone failed to decrease the size of the HIV-1 reservoir [164,165].

In an effort to elicit an HIV-1-specific CD8+ T cell response after latency reversal, one clinical trial administered a p24 peptide vaccine (Vacc-4x) prior to romidepsin treatment. This combination modestly but statistically significantly reduced the amount of total HIV-1 DNA and replication-competent virus [166]. Similar results were observed in another trial using a chimeric HIV-1 immunogen (HIVconsv) [167]. Combinations with the broadly neutralizing antibody 3BNC117, which targets the CD4 binding site on Env, have had variable results; while administration of romidepsin and 3BNC117 to newly diagnosed individuals showed a promising decrease in HIV-1 DNA [168], there was no significant decrease in the size of the reservoir when the same regimen was given to participants on LT-ART [169]. Altogether, these results emphasize the requirement for a strong anti-HIV-1 immune response in clearing the latent reservoir, which may actually be impaired by the use of HDACis in general [150].

### 3.2. PKC Agonists

**Figure 2:**
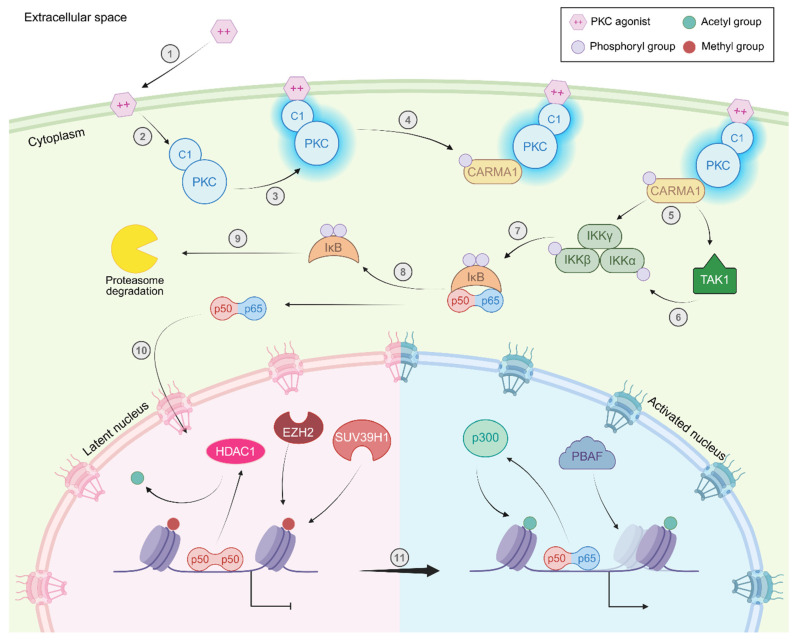
PKC agonists act through the NF-κB pathway. (**1**) PKCags mimic DAG in the plasma membrane. (**2**–**3**) PKCags bind to the C1 domain of PKC isoforms, which recruits them to the plasma membrane and enables them to take on an active conformation. (**4**) The active PKC can now phosphorylate and recruit CARMA1 to the site of the signalosome. (**5**–**6**) CARMA1, now active, recruits TAK1 and the IKK complex to the signalosome. TAK1 phosphorylates IKKα and IKKβ, which activates the complex’s own kinase activity. (**7**) The active IKK complex phosphorylates IκB, which normally binds and sequesters NF-κB p65 dimers in the cytoplasm. (**8**–**9**) IκB dissociates from p65/p65 and/or p50/p65 dimers. Now phosphorylated, it can be targeted for ubiquitination and proteasomal degradation. (**10**) p65 dimers translocate to the latent provirus. (**11**) p65 dimers bind the 5′ LTR, recruiting p300 and other pro-transcriptional factors to trigger full latency reversal.

Activation of protein kinase C (PKC) isozymes is a highly important step in a variety of cellular processes, as downstream signaling effects lead to the recruitment of TFs and epigenetic modifiers to genes involved in cell survival and proliferation, motility and adhesion, and even immune functions such as cytokine production (for a recent detailed review on this topic, see reference [170]). T cells, macrophages, and other cells in the HIV-1 reservoir express different PKC isozymes that activate distinct pathways towards cell activation, including the NF-κB pathway, through the recruitment of transcription factors and epigenetic modifiers to key genes (Figure 2) [170,171]; for the intents and purposes of this review, we will collectively refer to these isozymes as simply “PKC,” with further specification as necessary.

Previously, we reviewed the importance of NF-κB signaling in HIV-1 latency and reactivation (see reference [16]). In brief, the predominant NF-κB subunit in the nucleus of resting cells is homodimers of p50, which lacks a transcriptional activation domain; p65, by contrast, is sequestered in the nucleus by interactions with the IκB complex, which masks its nuclear localization signal (NLS) (reviewed in [172]). As we have discussed previously, p50 homodimers are also enriched at the latent provirus (Figure 1B and Figure 2), and are actually known to recruit class I HDACs that promote heterochromatin formation at the 5′ LTR (Figure 1B) [54]. During latency reversal, however, p65 homo- and p50/p65 heterodimers translocate to the nucleus and replace p50 homodimers at the 5′ LTR NF-κB binding site; here, they are presumed to recruit HATs that acetylate nuc-1 [106,109,173].

Under typical conditions, PKC is activated by diacylglycerol (DAG), which binds to the C1 domains of classical and novel PKC isozymes (Figure 2, step 2) (reviewed in [170]). As a result of this interaction, PKC relocates to the plasma membrane and takes on its active conformation (Figure 2, step 3) and phosphorylates the linker region of CARD-containing MAGUK protein 1 (CARMA1) (Figure 2, step 4), which allows them to associate with B cell lymphoma/leukemia 10 (BCL10), transforming growth factor beta-activated kinase 1 (TAK1, also known as MAP3K7) and the IκB kinase (IKK) complex at lipid rafts in the plasma membrane (Figure 2, step 5) [174,175,176,177]; this multi-enzyme assembly is sometimes referred to as the CBM signalosome [174,175]. With TAK1 positioned close to the IKK complex, it can phosphorylate the IKKβ subunit (Figure 2, step 6), which activates the complex to phosphorylate IκB and promote the release of p65 dimers (Figure 2, steps 7–8) (for a detailed review of NF-κB signaling regulation, see references [172,176]). With the NLS now exposed, free p65 dimers can translocate to the nucleus, bind to the 5′ LTR, and promote processive transcription from the provirus (Figure 2, steps 10–11). Notably, this process is facilitated primarily by PKC-θ in T cells and PKC-ε and -δ in macrophages (reviewed in [170]).

Small molecule PKC agonists (PKCags), such as phorbol 12-myristate-13-acetate (PMA, also called TPA in some sources), are structurally analogous to DAG and induce NF-κB signaling in a similar manner (Figure 2, steps 1–11) [178]. Indeed, early studies into epigenetic control of HIV-1 latency demonstrated that PMA stimulation significantly disrupts the chromatin environment of the 5′ LTR, destabilizing nuc-1 and increasing the accessibility of the TSS to transcriptional machinery [46,50]. Further studies into the mechanism behind this phenomenon revealed that PMA stimulation results in translocation of p65 to the 5′ LTR and hyperacetylation of both nuc-0 and nuc-1 (Figure 2, step 11) [110].

Importantly, however, PMA is also known to promote the formation of tumors, which is a clear limitation of its use as a therapeutic, in vivo LRA (reviewed in [179]). However, a number of cell-friendly natural products have been identified as having PKCag activity and have also been used in the context of HIV-1 latency reversal. In this section, we review the literature supporting a selection of PKCags’ use as LRAs.

#### 3.2.1. Prostratin

12-deoxyphorbol 13-acetate, more commonly known as prostratin, is a phorbol ester initially isolated from *Pimelea prostrata,* and has been the causative agent of many livestock poisonings in New Zealand [180]. Prostratin has since been isolated from members of the family Euphorbiaceae [181,182,183], and preparations of *Homalanthus nutans* and other species have traditionally been used in Samoa and other countries in the South Pacific to treat a variety of ailments, including back pain and diarrhea [181,182]. Notably, prostratin does not promote tumor formation, and in fact has been demonstrated to be protective against the effects of PMA in vivo [182,184,185]. In spite of this finding, in vitro studies have also shown that prostratin potently induces PKC activity. However, perhaps paradoxically, its affinity for PKC is low compared to other phorbol esters, such as phorbol 12,13-dibutyrate (PDBu) or 12-deoxyphorbol 13-phenylacetate (DPP) [182,184,186].

Early studies of prostratin’s effects in the context of HIV-1 infection revealed that multiple cell lines (CEM-SS, C1866, U937) were protected from virus-induced cytolysis and, in fact, experienced inhibited viral replication [182,187]. It was subsequently shown that prostratin decreased CD4, CCR5, and CXCR4 expression in a dose-dependent manner across multiple model systems, including PBMCs [187,188,189,190]. Indeed, pre-treatment with prostratin inhibited HIV-1 entry in PBMCs [188,189], suggesting a possible synergism with current ART regimens. At sub-protective doses, however, prostratin actually induced significant production of viral proteins and infectious virions in both lymphocytic and monocytic cells [182,186,187,191], including ex vivo-infected resting PBMCs [188,189]. In agreement with these findings, prostratin treatment also reactivated HIV-1 from latency in PBMCs from VS-PWH [190,192].

A notable difference between prostratin and the HDACis described previously is the capacity to induce the NF-κB pathway. In agreement with previous findings using PMA [178], prostratin treatment resulted in translocation of PKC-θ to the plasma membrane in J-Lat 6.3 cells, a Jurkat-derived line wherein the viral *nef* is replaced with a gene encoding eGFP [193]. This was unsurprisingly associated with increased IκBα phosphorylation and subsequent p65 recruitment to the latent LTR [191]. These results were corroborated in U1 cells, in which it was also observed that prostratin treatment led to nuc-1 acetylation and remodeling at the 5′ LTR [192], suggesting the recruitment of additional chromatin-modifying enzymes, particularly HATs and possibly PBAF. In accordance with the induction of the NF-κB pathway, prostratin also increased the expression of T cell activation markers CD69 and CD25 in resting PBMCs [188,189], which is another significant difference between prostratin and HDACis.

To this end, Jones et al. also found that prostratin sensitized infected CD4+ T cells to recognition by HIV-1-specific CD8+ T cells, but surprisingly inhibited the cytotoxic response [194]. In contrast with this finding, Desimio et al. observed that prostratin could not only modestly enhance NK cell activity, but also increase the susceptibility of infected CD4+ T cells to NK-mediated cytotoxicity, likely due to prostratin-induced modulation of receptor/ligand expression in either cell type [195].

Despite these differences in downstream effects, prostratin has been demonstrated to synergize well with HDACis. J-Lat and U1 cells treated with SAHA, TSA, or sodium butyrate in combination with prostratin experienced higher levels of LTR-driven transcription; nuc-1 was also more rapidly evicted from its canonical position after combined treatments, but histone acetylation was not increased above what was induced by HDACi treatment alone [192]. However, whether these combinations can induce a significant anti-HIV-1 immune response remains to be studied, and prostratin has yet to be utilized as an LRA in clinical trials.

To this end, one major limitation of further in vitro and in vivo studies of prostratin is its limited availability. Attempts at isolation from *H. nutans* have historically been unsustainable due to low and inconsistent yields [182]. However, Wender et al. have reported an economical synthesis of prostratin from phorbol, another natural product found in *Croton tiglium* seeds [196]. A key advantage of generating prostratin in this way is the ability to construct novel analogs de novo using an affordable, accessible starting material. Indeed, a series of analogs has been synthesized and shown to upregulate CD69 expression and downregulate CD4 expression, as does prostratin, albeit at significantly lower concentrations. Some analogs also stimulated transcription from the provirus in U1 cells and in PBMCs isolated from VS-PWH, even at nanomolar concentrations [197]. While further studies will be necessary to characterize these synthetic prostratin analogs and their effects on proteins that interact with the provirus, the ability to synthesize them from such a readily available natural source is promising for future in vitro and in vivo investigations.

#### 3.2.2. Bryostatin-1

Bryostatins are a family of natural macrolide lactones primarily isolated from the bacterial symbiont *Endobugula sertula,* of the marine bryozoan *Bugula neritina* [198]. All bryostatins consist of three pyran rings that together form the characteristic 20-carbon macrocyclic ring, which is critical for their binding to PKC. Of the bryostatins, bryostatin-1 (BRYO-1) is one of the most potent PKCags and has been demonstrated to have a variety of beneficial effects, including inhibiting tumor growth and invasion, reducing amyloid burden in murine models of Alzheimer’s disease, and improving symptoms of fragile X syndrome in mice (reviewed in [199,200]). While it is not yet commercially available, a number of clinical trials utilizing BRYO-1 have been completed as of the publication of this work [201].

In agreement with findings from other PKCags, BRYO-1 treatment has been demonstrated to downregulate expression of CD4, CCR5, and CXCR4 in multiple cell lines, as well as PBMCs, and inhibited viral replication [202,203,204,205]. However, in Jurkat and MAGI-HeLa cells, receptor expression was eventually restored despite sustained abrogation of new HIV-1 infections; likewise, VSV-G pseudotyped HIV-1 infection was also inhibited by BRYO-1 treatment, suggesting that the protective effect established by the compound was not entirely due to inhibition of viral entry [203].

In THP89 cells, which are derived from THP-1 cells but express GFP under control of the 5′ LTR [139], as well as the Jurkat-derived line J1.1 [206], BRYO-1 induced p24 production and GFP expression at nanomolar concentrations, and synergized with Tat-mediated transactivation [203]; similar results were observed in J89 cells, although BRYO-1 alone was less potent than in THP89 cells [207]. BRYO-1 could also induce viral gene expression in CD4+ T cells isolated from VS-PWH [204], and even reactivate HIV-1 from latency in infected astrocyte (NHA) and glial (U87) cell lines [208]. In contrast with these findings, BRYO-1 failed to induce virion production in primary monocyte-derived macrophages and microglia, and in fact impaired accumulation of mature Gag proteins, albeit by an unknown mechanism [202]. Taken together, these differences in results highlight the importance of considering cell type diversity when targeting the latent HIV-1 reservoir.

Unsurprisingly, BRYO-1 treatment was associated with increased activation of PKC-α and -δ in THP89 cells, and activation was abrogated when cells were treated with PKC inhibitors [203]. These results were corroborated in NHA, U87, and Jurkat-LAT-GFP cells [209], where BRYO-1 treatment also stimulated rapid p65 translocation to the nucleus [205,208], supporting the role of the NF-κB pathway in BRYO-1-mediated latency reversal. Interestingly, in one study, BRYO-1 failed to induce CD69 or CD25 expression in PBMCs [203], but significantly increased CD69 and CD38 expression in purified CD4+ T cells in another, especially when combined with panobinostat or romidepsin [207]. However, this difference may simply be due to differences in BRYO-1 concentration between the two studies, suggesting a dose-dependent effect on T cell activation.

In contrast with these in vitro and ex vivo findings, BRYO-1 failed to induce cell-associated or plasma HIV-1 RNA levels in PWH in a phase I clinical trial, nor did the tested dose induce PKC activity [210]. Unlike prostratin, BRYO-1 did not sensitize HIV-1-infected cells to NK-mediated cytotoxicity and, in fact, inhibited NK cell function [195]. To this end, BRYO-1 alone also attenuated the HIV-1-specific response of CD8+ T cells isolated from HIV-1 non-progressors (elite controllers), and actually caused exhaustion and death in these cells [211]. However, a recent study showed that BRYO-1 treatment decreased expression of exhaustion markers such as PD-1 and expanded HIV-1-specific CD8+ T cell populations when delivered with CD28 co-stimulation. These effects were attributed to stimulation of the p38 kinase MAPK11 [212], which can be activated through PKC signaling [213,214]. These contrasting studies once again underscore the importance of cell signaling not only in HIV-1-infected cells but also in cytotoxic cells proposed to target and eliminate the latent reservoir.

Similar to prostratin, BRYO-1-focused studies are hampered by the availability of the compound, as extraction from *B. neritina* is inefficient and unsustainable at scale, yielding only grams of product even from tons of starting material [215]. Chemical synthesis of BRYO-1 from accessible precursors has been successfully performed; in this process, the A and C rings are generated separately and combined by Yamaguchi esterification, which also generates the B ring [215,216].

Synthetic BRYO-1 is also advantageous over the natural product because it enables the generation of novel analogs, known as bryologs; indeed, Marsden et al. have found that bryologs reduced HIV-1 coreceptor expression on pCD4s at nanomolar concentrations and significantly suppressed viral spread in culture [217]. Several bryologs were also able to reactivate HIV-1 gene expression in J-Lat cells more effectively than BRYO-1 without inducing the production of inflammatory cytokines [217,218]. Another study further demonstrated that SUW133, a simplified analog of BRYO-1 that lacks the enolate carbonyl at C-13, could induce virion production in pCD4s isolated from VS-PWH. SUW133 additionally induced CD69 expression in uninfected CD4+ T cells. Importantly, SUW133 showed potent CD69 induction in humanized mice; when these mice were infected with HIV-1, treatment also triggered viral gene expression and productive infection. To this end, a modest number of HIV-1-infected cells died even without an additional HIV-1-specific immune response [219]. Synthetic bryostatins and their analogs are therefore a promising source of novel PKCags for HIV-1 latency reversal, as modifications to the base macrolide structure can be utilized to tweak PKC affinity and specificity, which may have differential effects on HIV-1 reactivation, T cell stimulation, and cytotoxic effects against infected cells.

### 3.3. Ingenoids

Ingenol is an ingenane diterpenoid that is structurally analogous to phorbol. Notably, the base ingenol molecule is not typically found in nature, and instead exists in various esterified forms within extracts of Euphorbiaceae family members (reviewed in [220]). Ingenol esters (collectively, ingenoids) have a wide variety of biological effects, including both tumor-promoting and anticancer activity, irritancy and inflammation, apoptosis, and growth factor promotion. These differences are in large part owed to functional group modifications at particular carbons on the ingenol base structure, particularly at C-3, -5, and -20 [221]. To this end, many ingenoids’ capacity to act as PKCags is dependent on the presence of a free hydroxyl group and a lipophilic group at any two of these positions, with C-3 serving as a common site for esterification to generate diverse ingenoids [220,221,222].

Ingenol 3,5,20-triacetate (ITA) was among the first ingenoids to be investigated for its activity in the context of HIV-1 infection. Its source plant, *Euphorbia kansui*, has been used in traditional Chinese medicine to treat a variety of ailments. ITA could cause significant downregulation of CD4 expression in MT-4 and MOLT-4 T cells and prevent new infections from occurring; however, treatment of OM10.1 cells, which are derived from HL-60 promyeloblasts and contain one provirus per cell [223], activated HIV-1 gene expression [224]. These findings are unsurprisingly in line with those we have discussed previously in this section. Another product of *E. kansui,* EK-16A, was also found to be a potent LRA in J-Lat, C11, and pCD4s, but was substantially more cytotoxic than prostratin at high concentrations. In contrast with many other PKCags, EK-16A exerted its activity through PKC-γ, and was more effective than prostratin in inducing p65 translocation [225].

Ingenol-3-angelate (I3A), also known as ingenol mebutate and PEP005, had been identified as an anticancer agent prior to investigation as an LRA [226], and has previously been available in the United States under the brand name Picato for the treatment of actinic keratosis [227]. In agreement with studies of other PKCags, I3A stimulation resulted in substantial downregulation of CD4 and CXCR4 expression in multiple cell types, including PBMCs, which had a protective effect against new infections. I3A could also induce viral gene expression from U1 and J-Lat A1 cells at nanomolar concentrations without inducing cell death [226,228], and was effective at inducing HIV-1 reactivation from pCD4s isolated from VS-PWH [228]; in one study, I3A could reactivate HIV-1 from all memory CD4+ T cell subsets [229]. Further investigation by Jiang et al. confirmed that I3A treatment induced phosphorylation of PKC-δ and -θ in J-Lat A1 cells, and promoted p65 binding to the LTR without upregulating expression of the protein. In accordance with this finding, I3A significantly upregulated the expression of T cell activation markers, but did not promote the production of pro-inflammatory cytokines [228]. Fascinatingly, topical application of Picato by PWH on ART-induced transcriptional initiation and production of full-length transcripts in not only peripheral CD4+ T cells, but also lymphocytes in skin biopsy samples from the participants, albeit to a more modest extent [230]. While further studies will be necessary to evaluate the efficacy and safety of I3A as an in vivo LRA, these initial results are promising towards the indication of ingenoids’ use in the “Shock and Kill” strategy.

In a study by Spivak et al. comparing a panel of synthetic ingenoids with varying modifications at C-3, some very interesting findings emerged. When a linear carbon chain was added to this position, saturated longer chain esters increased the compound’s LRA activity more than shorter chains; in particular, ingenol-3-hexanoate (IngB) and ingenol-3-octanoate (6- and 8-carbon chains, respectively) were more potent than ingenol-3-acetate and ingenol-3-butyrate (2- and 4-carbon chains, respectively). To this end, when branched chains or aromatic groups were added, complexity was positively correlated with potency. By contrast, introduction of unsaturated esters or polar groups reduced the efficacy of the compound, and additional esterifications at C-5 and C-20 abrogated the compounds’ LRA capacity. Of the ingenoids generated in this study, ingenol-3-acrylate was the most potent and could stimulate HIV-1 gene expression in pCD4s from VS-PWH to a very similar degree as TCR engagement [222]. While additional studies will be necessary to fully elucidate the effects of these compounds, this study provides valuable data towards the generation of novel synthetic ingenoids.

Ingenol 3,20-dibenzoate (IngDB), which was demonstrated to be less potent than other ingenoids featuring aromatic groups [222], has been shown to induce proviral transcription and CD69 expression in pCD4s from VS-PWH, and actually stimulated HIV-1 gene expression more consistently than panobinostat at the same dose. Notably, pCD4 activation was not associated with increased cell death. In further contrast to panobinostat, IngDB did not induce global histone H3 acetylation [231]; further studies will be necessary to determine IngDB’s effects on NF-κB signaling and proviral chromatin remodeling.

IngB, isolated from *E. tirucalli*, also potently reactivated HIV-1 in J-Lat A1 cells and pCD4s from VS-PWH even at sub-nanomolar concentrations [232,233]. Similarly to I3A, IngB induced p65 recruitment to the LTR, which was specifically associated with activation of PKC-δ [232]. A follow-up study demonstrated that IngB treatment also promoted translocation and activation of PKC-α, -δ, and -θ in HeLa cells, which was unsurprisingly associated with increased levels of p65 in the nucleus, and HIV-1 subtypes with multiple NF-κB binding sites were highly sensitive to IngB-mediated reactivation. However, IngB-mediated latency reversal was abrogated in cells expressing an LTR bearing a mutant NF-κB binding site [233], further supporting the notion that this pathway is the mechanism by which ingenoids activate HIV-1 gene expression. While IngB treatment induced CD69 expression in J-Lat A1 cells and primary PBMCs, it did not affect cell cycle proliferation or cytokine production [232]. These results were corroborated in another study that also demonstrated IngB’s ability to downregulate HIV-1 coreceptor expression and protect against new infections [234]. Notably, IngB was shown to be effective in vivo in virus-suppressed SIV-infected macaques, causing increased viral load in the plasma and cerebrospinal fluid of the animals as well as viral invasion into the brain tissue; however, this study did not investigate whether the compound could induce an HIV-1-specific immune response in vivo [235]. Interestingly, the base ingenol molecule is only a moderately effective LRA [233,236] and is unable to induce NF-κB activation in MOLT-4 cells [236]. Ingenol was also unable to significantly stimulate activity in any PKC isozyme [222], highlighting the necessity of the C-5 and -20 functional groups in binding and activating PKC. Importantly, however, ingenol is cytotoxic at concentrations required to reverse latency [233,237], suggesting that ingenoids may also act through pathways independent of PKC signaling.

As we have outlined here, nearly all ingenoids investigated as LRAs to date have been 3-O esters; however, one of the notable limitations of such compounds is their ability to undergo rapid ester migration, with each arrangement existing in equilibrium [238,239]. While this is a boon for developing new synthetic ingenoids, it also necessitates the development of other ingenoids that utilize different functional groups for stability and consistency. Indeed, Brehm et al. have reported the development of a novel ingenoid, GSK445A, where C-3 is connected to a carbamate group rather than an ester. GSK445A was demonstrated to be highly stable in culture over time, whereas IngB lost potency over the same period [240]. GSK445A could also induce HIV-1 gene expression in pCD4s from VS-PWH to a similar degree as IngB and PMA/ionomycin [240,241]. In accordance with results from other ingenoids, stimulation also induced CD69 expression and p65 translocation in pCD4s from VS-PWH; notably, it also promoted phosphorylation of P-TEFb, an important factor in processive transcription from the provirus. However, unlike the other LRAs we have discussed here, latency-reversing concentrations of GSK44A delivered to PBMCs from VS-PWH increased proliferation and interferon production in HIV-1-specific CD8+ T cells. Interestingly, GSK445A infusion into healthy rhesus macaques resulted in a rapid but transient decrease in peripheral T and B cells, NK cells, monocytes, and neutrophils, possibly due to invasion into tissues. These results were also observed in a preclinical study of SIV-infected macaques on ART, in which GSK445A treatment increased viral load in plasma and induced CD69 expression in both memory and naïve CD4+ T cells [241]. While this study did not evaluate GSK445A’s effects on SIV-specific CD8+ T cells, the ex vivo results paint a promising picture for the use of this novel ingenoid as a therapeutic LRA. To this end, the specific impact of the carbamate side chain remains to be investigated; the results of this study suggest that the use of this alternative functional group may promote both HIV-1 gene expression and CD8+ T cell function, but future studies will be required to elucidate the functional differences between ester and carbamate side chains in this context.

### 3.4. Other LRAs

As we have described in this work and others (see reference [16]), the transcription factors, enzymes, and structural proteins that regulate HIV-1 latency are diverse in their functions. While histone acetylation and NF-κB pathway activation are among the most-studied ways of activating proviral gene expression, other epigenetic factors and histone modifications have been investigated for the “Shock and Kill” strategy. In this section, we describe some of these unique LRAs and their targets.

#### 3.4.1. DNMT Inhibitors

As we have discussed previously, studies of in vitro HIV-1 latency models have demonstrated hypermethylation of CpG islands encompassing TF binding sites within the 5′ LTR and reactivation from latency upon treatment with DNMT inhibitors (DNMTis) [62,63,65,66,68,69,70,83,84,85]. These first-generation DNMTis, azacitidine and decitabine, have primarily been studied in the context of cancer chemotherapies and have received FDA approval for the treatment of myelodysplastic syndromes [242,243,244,245,246,247].

Azacitidine (5-aza-cytidine, 5-Aza-C), sold commercially as Vidaza [242,243,248], and decitabine (5-aza-2′-deoxycytidine, 5-Aza-dC), sold commercially as Dacogen [242,249], are cytidine analogs that inhibit methylation by DNMTs when incorporated into DNA (reviewed in [247,250,251,252,253]). The use of these drugs for HIV-1 latency reversal has demonstrated varying efficacies in proviral reactivation and altering methylation levels at the LTR [62,63,68,70,83,84,85,86,87]. Early studies demonstrated that azacitidine treatment resulted in demethylation of the HIV-1 LTR and reactivation in a T cell line containing an HIV-1 LTR reporter construct [62,63] and in ACH2 cells [68]. Interestingly, treatment with azacitidine did not impact NF-kB binding to the LTR in ACH2 cells [68], although it is thought that CpG methylation of the LTR likely contributes to latency by blocking TF binding [62,63,65]. However, other studies have demonstrated varied results across models, with enhanced reactivation of the provirus occurring when DNMTis are administered in combination with another potent activator [70,83,84,85,86,87]. One study using Sup-T1 cells containing a doxycycline-dependent HIV-1 construct reported greater reactivation using a combination of azacitidine and TNF-α versus either drug alone, suggesting an additive effect of demethylation [87]. Azacitidine in combination with lipopolysaccharide (LPS) strongly reactivated HIV-1-infected lymphocytes derived from transgenic mice compared with LPS stimulation alone [86]. Similar findings have been demonstrated in several J-Lat cell lines treated with a combination of decitabine and prostratin or TNF-α [70]. Another study found that decitabine combined with HDACis, PKC agonists, or TNF-α could overcome the inhibitory effects of hypermethylation in two J-Lat cell lines [83]. Furthermore, treatment with decitabine followed by administration of romidepsin or panobinostat in two J-Lat lines and in PBMCs isolated from PWH resulted in greater reactivation from latency compared with treatment of either inhibitor alone [85].

**Figure 3:**
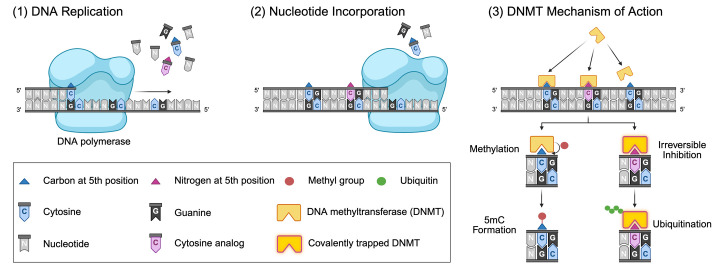
Mechanism of action for DNA methyltransferase inhibitors (DNMTi). (**1**,**2**) DNMTis such as azacitidine or decitabine are incorporated along with normal nucleotides during DNA replication. (**3**) Once DNA is synthesized, DNMTs can recognize and interact with the 5th carbon of cytosine within a CpG dinucleotide, facilitating the transfer of a methyl group at this position by nucleophilic attack, resulting in the formation of 5-methylcytosine (5mc). However, when a DNMT interacts with a cytidine analog, it becomes covalently trapped by interacting with the nitrogen atom substituted at the 5th position, resulting in irreversible inhibition and no methylation at this site. The covalently trapped DNMT is then eventually ubiquitinated for degradation.

The potential of these DNMTis for use in “Shock and Kill” strategies is complicated by two important factors. First, the requirement for nucleoside analogs to be incorporated into DNA in order for DNMT inhibition to occur (Figure 3) [76,254,255] is likely why HIV-1 reactivation under DNMTi treatment is only faithfully observed in cell lines, as incorporation would not occur in quiescent CD4+ T cells harboring an integrated provirus. Second, the findings of several studies in PWH on suppressive ART have shown generally low levels of methylation of the HIV-1 LTR relative to methylation levels observed in vitro [74,89,91,92,256,257]. It therefore seems that the limited efficacy of these drugs as LRAs is reflective of both a nuanced contribution of proviral methylation in latency maintenance and the biology of the latent reservoir.

Both azacitidine and decitabine have been approved for the treatment of myelodysplastic syndromes [242,243,244,245,246,247,248,249,250,258]. They are currently featured in many clinical trials for therapies targeting other myeloid malignancies along with other forms of cancer (336 and 168 trials in total for azacitidine and decitabine, respectively; in the active or recruitment phases [259,260]). These drugs are also being tested in trials for other conditions, such as cerebral palsy and graft-versus-host disease (azacitidine [259]), and COVID-19 and sickle cell disease (decitabine [260]). At the time of this writing, one clinical trial that will evaluate the administration of decitabine in combination with romidepsin for a “Shock and Kill” approach is in the recruitment stage [260,261].

#### 3.4.2. HMT Inhibitors

When compared with HDAC inhibitors, relatively few histone methyltransferase inhibitors (HMTis) have been discovered and developed for therapeutic use; therefore, only a few have been investigated as LRAs. Of particular interest to the field are inhibitors of SUV39H1, G9a, and EZH2, as these are the HMTs known to promote HIV-1 latency through methylation of H3K9 (SUV39H1 and G9a) and H3K27 (EZH2).

Chaetocin is a mycotoxin isolated from *Chaetomium* fungi and belongs to the larger family of epidithiodiketopiperazine complexes. It has a variety of anticancer effects, and has also been found to inhibit SUV39H1 by a nonspecific mechanism, likely by chemical modification of the enzyme [262,263]. When delivered to latently infected Jurkat T cells, J-Lat 15.4 cells, and pCD4s from VS-PWH, chaetocin induced HIV-1 gene expression in a dose-dependent manner but did not activate T cells [264,265]. In accordance with chaetocin’s target activity, treatment also led to decreased methylation of H3K9 on the LTR, which was also associated with increased acetylation on this residue [265]. To this end, combining chaetocin treatment with HDACis or PKCags had an additive effect on HIV-1 gene expression [264,265].

BIX01294 is a diazepin-quinazolin-amine derivative that was identified as a specific inhibitor of G9a via a high-throughput screen [266]. In early studies identifying the role of G9a in the context of HIV-1 infection, BIX01294 treatment induced HIV-1 gene expression in a dose-dependent manner in ACH2 and OM10.1 cells. To this end, H3K9 methylation was decreased at the LTR [267]. BIX01294 was also an effective LRA in pCD4s from VS-PWH [264] and, unsurprisingly, synergized with SAHA [264,267].

In contrast with these findings, a 2011 study by Friedman et al. demonstrated that neither chaetocin nor BIX01294 could induce high levels of gene expression in Jurkat E4 cells [58]. However, other studies have shown that H3K9 methylation may be less important than H3K27 methylation to maintain HIV-1 latency [60].

3-deazaneplanocin A (DZNep) is an analog of adenosine that was originally identified as an *S*-adenosylhomocysteine (AdoHcy) hydrolase inhibitor, and was later found to also inhibit EZH2—i.e., PRC2—by increasing AdoHcy levels in the cell [268,269], because AdoHcy inhibits methyltransferases (reviewed in [270]). In multiple Jurkat-derived cell lines, DZNep treatment alone induced HIV-1 gene expression, an effect that was enhanced by the addition of SAHA [58,60,271]. In agreement with previous findings, DZNep treatment also resulted in a loss of H3K27 methylation at the LTR [271].

Importantly, DZNep has been noted to have some toxicity in vitro when delivered at latency-reversing concentrations [58], possibly due to accumulation of AdoHcy [270], thus reducing its clinical potential. In contrast to DZNep’s mechanism of action, GSK343 binds to the *S*-adenosyl-L-methionine (SAM) pocket of EZH2 [272], thus increasing its specificity. GSK343 alone could modestly reactivate HIV-1 gene expression in infected primary Th17 cells [60]; however, in J-Lat A1, Jurkat 2D10, and U1 cells, it failed to induce gene expression beyond baseline levels [228,273]. However, GSK343 treatment did lead to a loss of H3K27 methylation at the LTR and sensitized cells to stimulation by SAHA [273]. Taken all together, these studies suggest that inhibition of HMTs alone may not always be sufficient to reactivate HIV-1 gene expression, and that addition of other compounds to promote euchromatin formation may be necessary to “shock” HIV-1 out of latency.

#### 3.4.3. Bromodomain Inhibitors (LRA)

As we have discussed previously, nucleosome positioning and related factors are important in regulating latency. BRD4 recruits the BAF complex to nuc-1 to maintain its repressive position, which is inhibitive to proviral gene expression [47,48]. JQ1 is a thienodiazepine compound that binds in the acetyl-lysine pocket of BRD4 (see reference [274] for an in-depth review of bromodomain structure), thus displacing it from acetylated histones and other proteins [275]. In normal cellular genes, this is inhibitive to transcription because BRD4 usually recruits P-TEFb to promoters. When delivered to J-Lat A2 and Jurkat 2D10 cells, however, JQ1 increased GFP expression in a dose-dependent manner, suggesting that it actually promotes HIV-1 gene expression [276].

Further studies revealed that JQ1 accomplishes this through a couple of different mechanisms. First, JQ1 enhanced Tat’s activity by dissociating BRD4 from P-TEFb, thus relieving competition [276]. Second, JQ1 treatment displaced the BAF complex from the 5′ LTR, which was accompanied by increased DNA accessibility at this region. However, this effect was independent of transcriptional initiation [48]. To this end, JQ1’s potential for latency reversal has been found to be improved when delivered with compounds that promote transcription from the provirus, particularly PKCags but also PAF1C inhibitors [228,276,277,278].

Another bromodomain inhibitor, OTX015 (also known as birabresib), has also been found to stimulate GFP expression in J-Lat C11 cells in a Tat-dependent manner, but its effective concentration was much lower than that of JQ1 [279]. Interestingly, a novel inhibitor of BRD9 could also induce HIV-1 gene expression in J-Lat cells and pCD4s from VS-PWH [280]. While bromodomain inhibitors have not yet been investigated for their use as LRAs in vivo, oral administration of OTX015 has been well-tolerated in clinical trials [281]. Because bromodomain inhibitors are enhanced when combined with other latency-reversing compounds, coadministration may be an effective strategy to increase the potency of current LRAs.

#### 3.4.4. Inhibitors of Nucleosome Remodeling Complexes

Unsurprisingly, the BAF complex itself has been targeted for latency reversal. Pyrimethamine, an antimalarial drug, and caffeic acid phenylethyl ester (CAPE) were identified as having inhibitory activity against BAF in a screening [282]. These small molecules were able to reactivate HIV-1 gene expression in J-Lat A2 and 11.1 cells, induced a similar gene expression profile to BAF250a knockdown, and unsurprisingly displaced the BAF complex from nuc-1, although at somewhat high concentrations. Pyrimethamine and CAPE also synergized well with SAHA and prostratin, but did not induce T cell activation when delivered alone [283]. In a recent clinical trial, pyrimethamine was able to induce HIV-1 transcription in VS-PWH, but it did not reduce the size of the latent reservoir [284].

The macrolactam BRD-K98645985 (BRD-K) was similarly identified in a screen of inhibitors of BAF250, a subunit that is exclusively found in the BAF complex. BRD-K could induce HIV-1 gene expression in J-Lat 11.1 cells and pCD4s from VS-PWH, albeit at high concentrations. Notably, BRD-K did not induce T cell activation, but it did alter the expression of some cellular genes that are regulated by the BAF complex; however, at its effective concentration, cell viability was not impacted by treatment [285].

Another class of small molecules, curaxins, is known to inhibit the FACT complex, which has been implicated in HIV-1 latency—although its exact role remains unclear (reviewed in [16]). Curaxins are unique among the small molecules discussed here in that they intercalate nucleosomal DNA, causing torsional stress and nucleosome disassembly that recruits FACT complexes and causes them to bind tightly to DNA. Normally, this is repressive to transcription as FACT complexes become “trapped” at these destabilized nucleosomes [286,287,288]. In contrast with this, the curaxin CBL0137 (also called CBLC137 in some literature) has been identified as a potential LRA. In pCD4s from VS-PWH, CBL0137 could induce HIV-1 gene expression to similar levels as TCR stimulation; however, in the cell line models J-Lat 6.3 and CA5, treatment with the curaxins alone was insufficient for latency reversal. The authors postulated that this discrepancy may be due to nonfunctional p53 pathways in Jurkat-derived cell lines, which may otherwise promote HIV-1 reactivation in PWH [289]. Notably, CBL0137 has also been found to promote proviral integration, likely due to global chromatin opening [11].

Altogether, these studies highlight the potential for targeting nucleosome positioning and stability for latency reversal. However, because nuc-1 remodeling occurs prior to and is considered to be independent of transcription from the provirus, it is likely that these small molecules will require coadministration with other LRAs such as PKCags to achieve high levels of HIV-1 gene expression.

#### 3.4.5. Tat as an LRA

While the small molecules and chemical agents described herein primarily target host epigenetic and transcriptional regulation, HIV-1 Tat has been investigated for its potential as an HIV-specific latency reversal agent [290,291]. Studies on exogenous Tat delivery have demonstrated potent latency reversal [291,292,293,294,295], but concerns regarding associated cytotoxicity, cellular activation, and in vivo stability have been reported [290,296,297,298]. However, more recent studies have evaluated the use of nanoparticles, such as exosomes and lipid nanoparticles (LNPs), for the delivery of Tat for efficient reactivation of the latent provirus while minimizing such effects.

Initial studies on Tat delivery to latently infected cells via nanoparticles began with an exosome delivery system (EXO-Tat) to deliver the full-length Tat protein. The administration of EXO-Tat in CD4+ T cells derived from individuals on LT-ART exhibited limited potential for latency reversal when compared to reactivation under PMA/ionomycin stimulation; however, a combination of EXO-Tat with either panobinostat or disulfiram synergized to significantly reactivate latent proviruses relative to controls [299]. While EXO-Tat did not result in T cell activation or increased levels of pro-inflammatory cytokines [299], a subsequent study revealed that EXO-Tat administration significantly altered the host cell proteome [300].

Alternatively, a handful of recent studies have demonstrated success in reversing HIV-1 latency while minimizing cytotoxicity and global T cell activation through the use of LNPs for Tat mRNA delivery [301,302,303,304]. In one study, researchers identified a truncated Tat variant that effectively reactivated HIV-1 in both latently-infected Jurkat cells and in CD4+ T cells from cART-treated individuals [304]. Moreover, they demonstrated that reactivation efficiencies were enhanced when delivering Tat mRNA via an LNP system when compared to the delivery of exogenous Tat protein alone [304]. Another report found that using the same delivery system (Tat-LNP) in combination with Panobinostat significantly induced HIV-1 reactivation from CD4+ T cells derived from VS-PWH when compared to either treatment alone or with PMA/ionomycin stimulation [302]. Notably, delivery of Tat-LNPs alone did not result in cellular activation or transcriptomic alterations in the cell lines or pCD4s used in these studies, which is important given the known ability of Tat to alter cell function [302,304]. In another study, the authors demonstrated that Tat-LNP delivery resulted in HIV-1 latency reversal in a TAR-dependent manner with varying degrees of reactivation across five latent cell line models, and induced HIV-1 transcriptional elongation, polyadenylation, and splicing in CD4+ T cells from VS-PWH [303]. Tat-LNP delivery was also shown to synergize with other LRAs, namely AZD5582, SAHA, and I-BET151, to reactivate HIV-1 across the majority of donors tested [303]. Recently, another study reported the use of a different LNP system (LNP X) to deliver Tat mRNA to resting CD4+ T cells from VS-PWH, without the need for stimulation prior to transfection [301]. Taken together, these studies offer support for the continued investigation of Tat-LNP systems in the development of an HIV-1-specific latency reversal strategy.

## 4. Block and Lock

With the current limitations and challenges of Shock and Kill, it is necessary for alternative cure strategies to be proposed and investigated as well. “Block and Lock” is one such strategy, and rather than clearing the latent provirus, its strategy aims to use latency promoting agents (LPAs) to force the provirus into a state of “deep latency” from which it cannot easily reactivate.

Several studies have focused on using LPAs to inhibit the assembly and recruitment of transcriptional machinery to “lock” HIV-1 into deep latency [305,306,307,308]. However, as we have discussed previously, sequestration of certain transcription factors like NF-κB p65 and formation of heterochromatin are also highly inhibitive to efficient proviral gene expression. Indeed, just as the epigenetic factors that promote HIV-1 latency can be targeted by small-molecule agonists and inhibitors, some LPAs can promote a transcriptionally repressive chromatin environment at the 5′ LTR. In this section, we review the literature focusing on a selection of LPAs known to affect chromatin on the integrated provirus. Because this is not an exhaustive list of all LPAs under investigation, the exclusion of any particular LPA is in no way meant to serve as commentary on its therapeutic or scientific potential.

### 4.1. Tat Inhibitors

As we have discussed previously, the expression and activity of Tat are highly important to productive HIV-1 infection. It is well-understood that Tat recruits P-TEFb to the 5′ LTR, leading to phosphorylation and processive activity of RNAPII. Tat additionally associates with other pro-transcriptional factors, such as p300/CBP [309]. Taken together, these factors make Tat an attractive target for LPAs, as we describe in this section (summarized in Table 2).

#### 4.1.1. Nullbasic

Nullbasic is a full-length (that is, two-exon) engineered mutant form of Tat wherein the entire basic domain is replaced with glycine and alanine residues (Table 2), which causes Nullbasic to become localized to the cytoplasm as opposed to the nucleus [324]—an effect that has been seen with other basic domain mutants [325]. Indeed, other Tat mutants where the basic domain is absent (as in one-exon mutants) or otherwise disrupted have been demonstrated to have a transdominant negative phenotype against wild type Tat [325,326,327]. In accordance with these results, Nullbasic could potently inhibit viral replication across multiple cell types and models of infection, including pCD4s [310,311,314,324], and importantly has been shown to be effective against HIV-1 subtypes B, C, and D [328]. Additional studies revealed that Nullbasic exerts this suppressive function through several mechanisms.

First, Nullbasic seems to disrupt shuttling and nucleolar localization of the viral protein Rev [324]. Briefly, Rev is normally imported to the nucleus through interactions with transportin, importin proteins, and nucleolar phosphoprotein B23 (B23) [329]. Within the nucleus, it binds to the Rev response element (RRE) of incompletely spliced viral mRNAs; this may be facilitated by DEAD-box RNA helicase 1 (DDX1)’s remodeling of the RRE to promote Rev interaction. Chromosomal region maintenance gene 1 (CRM1) and RanGTP interact with its nuclear export sequence (NES), enabling its return to the cytoplasm and release of viral mRNAs for translation (for a detailed review on the structure and function of Rev, see reference [330]). In the presence of Nullbasic, however, Lin et al. found that the Rev/CRM1 and Rev/B23 complexes were disrupted, but the localization of CRM1 and B23 was unaffected when Nullbasic was expressed alone. This effect was abrogated when Rev’s NES was mutated such that it could no longer interact with CRM1 [312]. Nullbasic also re-localized DDX1 to the cytoplasm, which caused Rev to become trapped in the nucleus [313]. Taken together, these results support the notion that Nullbasic promotes HIV-1 latency by disrupting viral mRNA export.

In addition to altering viral mRNA export, Nullbasic suppresses transcription from the provirus. No viral mRNA was detected in HIV-1-infected Jurkat T cells expressing Nullbasic (NB-Jurkats) [310,311]. Additionally, proviruses in NB-Jurkats and J-Lat 6.3 cells were resistant to reactivation after stimulation with PMA or JQ1. Importantly, this effect was independent of cellular activation, as CD69 and CD25 expression were upregulated after PMA treatment. Proviruses in NB-Jurkats were also hypoacetylated at H3K9 on nuc-1 [310], which is in agreement with the observation that wild-type Tat recruits p300/CBP through its basic domain [309]. As such, this interaction is expected to be absent in mutants such as Nullbasic. To this end, treating NB-Jurkats and J-Lat 6.3 cells with SAHA rescued gene expression from the provirus [310], indicating Nullbasic suppresses HIV-1 gene expression by both blocking transcription from the provirus and disrupting translation of mRNAs that are produced.

Interestingly, it has also been observed that Nullbasic inhibits viral replication by reducing the infectivity of virions [310,324]. Indeed, further coimmunoprecipitation experiments revealed that Nullbasic associates directly with RT [315], which is in agreement with previous findings that wild-type Tat also interacts with and stimulates RT [331]. However, while Nullbasic was unable to inhibit RT activity in in vitro assays, it did disrupt reverse transcriptase complex (RTC) formation in virions from HIV-1-infected HEK293T cells, specifically altering the distribution of capsid proteins; to this end, viral cores from Nullbasic-expressing cells were more sensitive to disassembly [315].

In agreement with these in vitro findings, treating pCD4s with Nullbasic prior to transplantation into NSG mice strongly inhibited HIV-1 infection in the periphery and tissues. When mice were treated after infection, Nullbasic both delayed viral replication and suppressed expansion into the organs; additionally, it protected CD4+ T cells from the cytopathic effects of acute infection, an effect that persisted even after viral rebound [314]. Taken all together, these in vitro, ex vivo, and in vivo studies indicate that Nullbasic is an effective agent to suppress HIV-1 gene expression and expansion, and exerts this effect through a number of different mechanisms.

What remains to be determined, however, is how Nullbasic behaves in more clinically relevant models. The current in vivo study utilizes mice that have received pCD4 grafts, rather than central nervous system (CNS) grafts or the EcoHIV model that is known to establish latent reservoirs in multiple tissue types [332]. Therefore, this model may not fully recapitulate the minutiae of HIV-1 spread and pathology, particularly in the CNS. Indeed, wild-type Tat is produced by HIV-1-infected cells in the CNS and excreted to the extracellular space, which contributes to neuroinflammation and aberrant gene expression in nearby cells, ultimately contributing to the development of HIV-1-associated neurocognitive disorders (HAND) (see references [298,333,334,335] for some of our group’s previous work on this topic). Due to the limited number of in vivo studies of Nullbasic’s functions, it remains to be determined whether it can ameliorate these effects or how it interacts with ART. If Nullbasic can, in fact, reduce neuroinflammation and resolve HAND symptoms, it may be a highly effective adjuvant to current ART regimens.

#### 4.1.2. Didehydro-Cortistatin A

Cortistatins are a group of steroidal alkaloids isolated from the marine sponge *Corticium simplex.* Of these compounds, cortistatin A (CA) has been demonstrated to be a potent inhibitor of growth in human umbilical vein endothelial cells (HUVECs), Neuro2A cells, and others [336], and is a high-affinity ligand and inhibitor of CDKs 8 and 11 [337]. CDK11 has been implicated in 3′ end processing in HIV-1 mRNAs [338], and is therefore an attractive target for the “Block and Lock” strategy. However, the scalability of studies and the use of CA are limited by its scarcity in the environment, necessitating the development of a synthetic pathway utilizing accessible materials. Indeed, Shi et al. have proposed such a pathway that produces large quantities of CA using the common medicinal steroid prednisone as the starting material [339].

Didehydro-cortistatin A (dCA) is the immediate precursor to CA in this synthetic pathway and, as such, is highly structurally similar to CA (Table 2), but was found to be equivalent to CA as an inhibitor of HUVEC growth [339,340]. In addition, dCA could inhibit transactivation and transcriptional elongation in productively infected HeLa-CD4 cells, but had no effect when cells were stimulated with PMA or TNF-α [316]. Further, knocking down CDK8 did not affect dCA’s inhibitory activity [317]. Taken together, these studies suggest that dCA’s inhibition of HIV-1 gene expression is Tat-dependent. Indeed, pulldown of biotinylated dCA also precipitated Tat, except when the basic region was mutated [316]; additional studies revealed that dCA directly interacts with this domain, blocking its interaction with the TAR while stabilizing Tat’s structure [317].

In agreement with these findings, treating latently infected HeLa-CD4 cells with dCA led to sustained and progressive suppression of HIV-1 gene expression, which was enhanced in the presence of ART [318,319]. dCA also further suppressed transcription in the latently infected cell lines J-Lat 6.3 and 10.6, and OM10.1, as well as pCD4s from VS-PWH, and protected latently infected cells from reactivation with PMA, TNF-α, and SAHA [318]. However, dCA resistance developed over time after infected cells were cultured in the presence of increasing doses of the compound. In particular, these viruses had higher baseline levels of transcriptional activity due to enhanced NF-κB-mediated activation; interestingly, no mutations in Tat or the TAR were noted [320], further supporting dCA’s Tat-dependent mechanism, but also suggesting that dCA alone may be insufficient to fully “lock” HIV-1 proviruses into deep latency.

Despite this limitation, that dCA is otherwise able to induce long-term viral suppression and prevent reactivation in latent proviruses suggests a potential role of epigenetic control. Indeed, in the presence of ART, dCA treatment increased protection from MNase digest at the 5′ LTR, as well as increased H3 levels at nuc-1 [319,321], suggesting tighter interactions between the 5′ LTR nucleosomes and their associated DNA. To this end, treatment also induced a significant decrease in H3K27ac levels at nuc-1; the 5′ LTR also experienced a loss of PBAF and increased occupancy of BAF [319]. These results are not surprising due to the role of Tat’s recruitment of p300/CBP and PBAF to the 5′ LTR [47,112,114,309].

Critically, dCA has also been demonstrated to be effective in the astrocyte-like line U87MG. In vitro, Tat stimulated these cells to express pro-inflammatory cytokines like IL-1β and TNF-α, but dCA treatment significantly reduced this effect. Further, administration of dCA in vivo ameliorated cocaine-seeking behavior in conditioned, Tat-expressing transgenic mice; Tat alone, by contrast, enhanced the behavior [322]. In agreement with previous in vitro findings, dCA treatment significantly reduced both viral mRNA production in HIV-1-infected, humanized mice and was able to control rebound viremia and viral gene expression after ART cessation. Notably, this effect was seen in the periphery and tissues, including the brain [321].

Taken all together, these studies highlight the therapeutic potential of dCA in not only long-term control of HIV-1 gene expression and replication, but also treating CNS infection and even alleviating some of the effects of HAND. However, for effective usage as an adjuvant or replacement for ART, dCA will have to be formulated such that single-dose or short-term administration has lasting effects. Additionally, as dCA can also act on CDKs 8 and 11 [339], the in vivo effects of this interaction will need to be carefully evaluated for safety and quality of life impact, especially when compared with current ART regimens.

#### 4.1.3. Triptolide

Triptolide is a diterpenoid epoxide isolated from the Chinese vine *Tripterygium wilfordii Hook F* (Table 2) and has a broad range of bioactivities, including immunomodulation and inhibition of tumor cell proliferation [341]. Triptolide has also been found to disrupt the formation of super-enhancer networks in pancreatic fibroblasts [342]. However, it has been noted for its toxicity in multiple organs (reviewed in [343]) and is mostly insoluble in water [344], thus limiting its bioavailability. To overcome these challenges, the Saluja group at the University of Minnesota developed Minnelide, a prodrug of triptolide with greater antitumorigenic effects [344]. Minnelide has since been the subject of a clinical trial to treat adenosquamous carcinoma of the pancreas [345].

Triptolide’s suppression of HIV-1 infection was identified in TZM-bl cells, a HeLa-derived line that expresses luciferase under control of the 5′ LTR but requires exogenously supplied Tat or infection with full-length HIV-1 [346], where it potently suppressed viral replication and gene expression without reducing cell viability. These results were corroborated in Jurkat T cells and ex vivo-infected PBMCs. However, triptolide did not affect basal transcription of the provirus, nor did it prevent TNF-α-mediated reactivation [323]. Taken together, these results suggest that triptolide’s latency-promoting mechanism is Tat-dependent.

Indeed, the levels of Tat protein in triptolide-treated cells were significantly lower than in untreated cells. However, Tat transcripts were not significantly affected [323], suggesting a post-translational inhibitory mechanism. Addition of the proteasome inhibitor MG132 rescued Tat protein expression, indicating that triptolide suppresses proviral transcription by promoting proteasomal degradation of Tat, and it was further demonstrated that this effect requires Tat’s acidic, cysteine, and core regions to be intact [323]. This is in contrast to Nullbasic and dCA, which rely on the basic domain for their latency-promoting activity, as we discussed previously in this section. However, the mechanism by which triptolide marks and targets Tat for degradation is not yet known.

While the epigenetic effects of triptolide treatment at the 5′ LTR have not been elucidated, it is likely that it promotes suppressive chromatin at the proviral promoter by preventing the recruitment of latency-reversing factors by Tat. Additionally, triptolide has been found to increase lysine-specific histone demethylase 1A (LSD1) expression in multiple myeloma (MM) cells, leading to demethylation of H3K4 and preventing transcription [347]. Interestingly, another study demonstrated that triptolide decreased H3K9 and H3K27 methylation in a dose-dependent manner, along with expression of their respective methyltransferases [348]. Altogether, these studies suggest that triptolide could induce heterochromatin formation at the 5′ LTR, but further studies will be required to confirm this, as well as triptolide’s effects on other cell types found in the reservoir.

### 4.2. Transcriptional Gene Silencing

**Figure 4:**
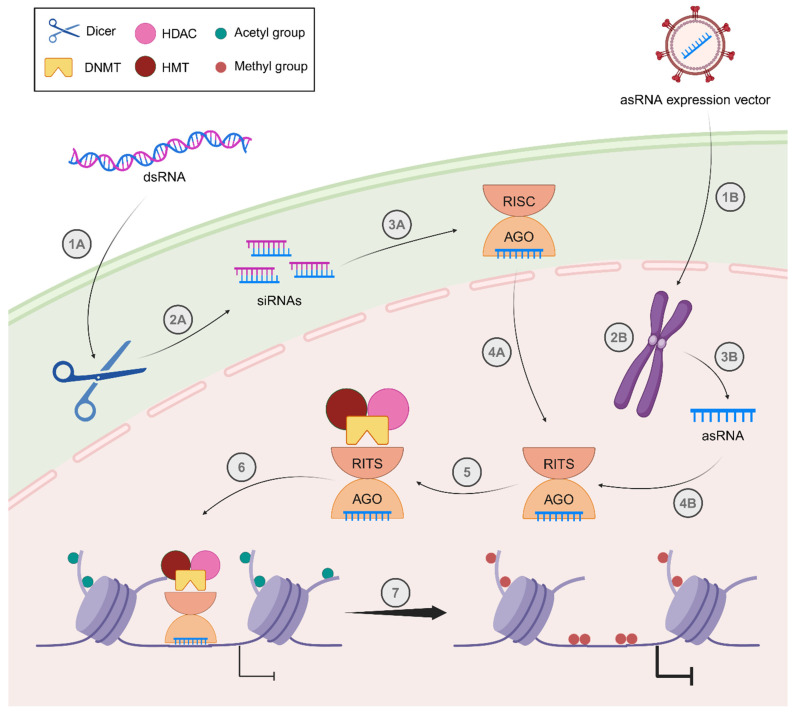
Pathway of transcriptional gene silencing. (**1A**) Exogenously produced dsRNAs, such as viral genomes, are delivered to the cytoplasm. (**1B**) Therapeutic vectors such as lentiviruses can also deliver dsRNA and asRNA expression vectors for endogenous synthesis and processing. (**2A**) Dicer processes dsRNAs into siRNAs. (**2B**) siRNA vectors are delivered to the nucleus; in the case of lentiviral constructs, they integrate into the genome. (**3A**) One strand of the siRNA is loaded into RISC in the cytoplasm. (**3B**) Single-stranded RNAs, such as asRNAs, are expressed by the host cell or vector. In the case of dsRNAs, they are processed by nuclear-localized Dicer. (**4A**) RISC translocates to the nucleus, where it becomes the RITS complex. (**4B**) Single-stranded RNAs are loaded into the RITS complex. (**5**) The RITS complex assembles with repressive factors, namely DNMTs, HMTs such as EZH2, and HDACs. (**6**) The RITS complex is guided to the genomic region complementary to its loaded siRNA. (**7**) The factors associated with the RITS complex act on their substrates. 5′ LTR nucleosomes are thus deacetylated and hypermethylated, and the CpG islands are also methylated.

Transcriptional gene silencing (TGS) is a mechanism of transcriptional control that occurs at the level of the genome. TGS has long been recognized in plants, yeast, and invertebrate animals, in which cytoplasmic double-stranded RNAs (dsRNAs)—such as viral genomes—are processed by the endoribonuclease Dicer (in plants, Dicer-like) to form small interfering RNAs (siRNAs) and microRNAs (miRNAs) (Figure 4, steps 1–2A) (reviewed in [349,350,351]). siRNAs are loaded into the RNA-induced silencing complex (RISC), the key components of which are Argonaute (AGO) proteins that associate with the siRNA directly (Figure 4, step 3A). The loaded RISC can then translocate to the nucleus, where it assembles with DNA methyltransferases and histone-modifying enzymes to form the RNA-induced transcriptional silencing (RITS) complex (Figure 4, steps 4A–5) (reviewed in [350,352,353]). dsRNA molecules may also be transcribed from the genome—for example, when delivered by a lentiviral vector (Figure 4, steps 1–2B)—processed by nuclear-localized Dicer to siRNA, and loaded onto the RITS complex (Figure 4, steps 3–4B) (reviewed in [354,355]). The siRNA binds to its complementary sequence on the genome, bringing the RITS complex close to its histone and DNA substrates, thus inducing heterochromatin formation and gene downregulation (Figure 4, steps 6–7) (reviewed in [351,353,354]). A classic example of TGS in mammals is X-chromosome inactivation, whereby Xist RNAs coat one of the X chromosomes at random, promoting the formation of repressive chromatin and DNA methylation that potently suppresses expression of nearly all genes (reviewed in [356]).

Altogether, it would stand to reason that TGS could be an effective method to control the expression of aberrant genes. Indeed, in a study by Hawkins et al., it was demonstrated that an shRNA targeting the promoter of *UbC*, which encodes ubiquitin, significantly downregulated expression of the gene in HEK293-derived cells. In agreement with this finding, the *UbC* promoter was hypermethylated. However, *UbC* expression could be rescued by treating the cells with TSA, azacitidine, or a phosphorothioate oligodeoxynucleotide (ODN) complementary to the promoter-associated strand of the shRNA. Importantly, the shRNA-induced *UbC* knockdown lasted over a month, and additional experiments revealed that DNA methylation was largely responsible for this effect, while histone hypermethylation and hypoacetylation were mostly dispensable [357].

That TGS could induce such potent and stable suppression of *UbC* is highly intriguing from a therapeutic perspective. As we have discussed previously, the epigenetic patterns induced by TGS are highly important to maintaining the latent state of the HIV-1 provirus. Therefore, TGS may present an opportunity to both “block” HIV-1 transcription and “lock” the provirus into the highly desirable state of deep latency. In this section, we describe some of the small noncoding RNAs that have been designed and investigated as part of the “Block and Lock” strategy.

#### 4.2.1. PromA

The sequence of PromA is identical to the NF-κB binding site on the NL4-3 LTR, about 100 nucleotides upstream of the TSS. The siRNA form, siPromA, was one of the earliest attempts at using TGS to suppress HIV-1 gene expression. Virion production in HIV-1-infected MAGIC-5 cells, a HeLa-derived line expressing CCR5/CXCR4 and CD4 [358], was nearly abrogated, as was proviral transcription, in cells treated with siPromA [359]. In agreement with previous findings, siPromA treatment induced significant methylation of the 5′ LTR specifically, which was lost when the cells were treated with azacitidine [359]. Likewise, siPromA treatment led to enrichment of HDAC1 at the 5′ LTR, as well as hypermethylation of H3K9 at nuc-0 and nuc-1. Interestingly, the repressive positioning of nuc-1 was also stabilized near the TSS [360].

The short hairpin RNA (shRNA) form of PromA (shPromA, also called shκB in some studies) yielded similar results in HIV-1-infected MOLT-4 T cells. When the shRNA was delivered by a retroviral vector, it could potently suppress HIV-1 replication for over a year [361]; these results were further corroborated in HIV-1-infected PBMCs, where any viral outgrowth was attributed to cells that did not receive the vector [362]. Retroviral delivery also led to a progressive increase in H3K9 and H3K27 methylation at the 5′ LTR; to this end, TSA could rescue HIV-1 gene expression in HeLa cells expressing luciferase under control of the 5′ LTR (HeLa/LTR-luc), and PM1-CCR5 cells, but the reactivation potential was much lower than in cells treated with a control shRNA [361,362]. Interestingly, shPromA did not induce significant levels of DNA methylation at the 5′ LTR [361], possibly due to differences in cell type between the studies, as shPromA was also found to be processed into the siRNA form via cleavage of the loop structure [363].

In agreement with these in vitro findings, delivery of shPromA-transduced PBMCs to NOJ mice resulted in significantly lower viral loads compared to control mice; likewise, treated mice had a higher CD4+ T cell count. Specifically, expression of the antisense strand of the shRNA was strongly inversely correlated with viral load and cell-associated *gag* mRNA. While PMA could rescue HIV-1 gene expression ex vivo [362], it is unknown how in vivo delivery of LRAs would affect viral load when administered to shPromA-treated mice. However, given that proviruses in elite controllers are often found in regions of tight heterochromatin, it is likely that shPromA could still effectively control the infection under in vivo latency reversal conditions, especially when delivered with long-acting ART.

One of the potential challenges of utilizing TGS to control HIV-1 expression is the diversity of quasispecies that exist in PWH, both between and within individuals, necessitating the use of small RNAs that target conserved regions. The NF-κB binding region is well conserved between quasispecies and HIV-1 subtypes [364], and indeed siPromA was demonstrated to be able to suppress viral replication across multiple laboratory strains. However, neither it nor shPromA had a significant effect on HIV-2 [359,363]; this is not surprising, as HIV-2’s NF-κB binding region is distinct from HIV-1’s [365]. To this end, mismatches deliberately introduced into the sequence of shPromA eliminated the shRNA’s ability to suppress HIV-1 replication. Importantly, shPromA did not alter expression of cellular NF-κB-dependent genes, nor did it seem to have off-target effects [363]. Taken together, these results suggest broad-spectrum but HIV-1-specific efficacy of PromA, but additional studies will be required to determine if it can suppress other HIV-1 subtypes.

#### 4.2.2. LTR-247 and LTR-362

LTR-247 and LTR-362 were developed as part of an investigation into the relative efficacy of the RNA duplex, antisense strand, and sense strand to mediate TGS. LTR-247 targets a site slightly downstream of nuc-0, whereas LTR-362 overlaps PromA except for a few nucleotides. In this study, it was found that delivery of the antisense strand alone (247as and 362as) was highly effective in reducing luciferase expression in 1G5 cells [366], a Jurkat-derived line that expresses luciferase under control of the 5′ LTR when Tat is present [367].

In a follow-up study, it was demonstrated that, when delivered via an HIV-2-based mobilization-competent vector (MCV)—which requires HIV-1 infection to supply the necessary factors for gene expression and replication (reviewed in [368])—362as could suppress luciferase expression in TZM-bl cells in the presence or absence of Tat, likely due to its position over the NF-κB binding site. Indeed, 362as completely blocked p65 from binding [369], possibly due to steric interference by AGO1. Both RNAs could suppress HIV-1 gene expression in infected Jurkat T cells; for cells treated with 362as, the infection was controlled for nearly a month. In agreement with these and other previous findings, 247as and 362as treatment led to increased methylation of H3K27 at the LTR; to this end, 362as treatment recruited DNMT3a and HDAC1 to the LTR [369].

Notably, 362as was more effective in the presence of AZT [370], supporting the notion that TGS could support current ART regimens. However, this RNA was cytotoxic in pCD4s, an effect that was attributed to off-target effects against the small nucleolar RNA, *SNORD45b*, which aids in ribosomal RNA methylation. Blocking this activity could induce ribosomal stress, and indeed, p53 was upregulated in 362as-expressing cells. Additionally, cells treated with the shRNA form 362sh showed suppressed population growth. The authors of this study postulated that the high MOI required to achieve the antiviral effects of 362as or 362sh may have oversaturated the cells, thereby inducing harmful effects via multiple pathways [370].

However, when delivered exogenously via a gp120 aptamer, the dsRNA form of LTR-362 was effective in suppressing HIV-1 gene expression in pCD4s with no notable changes in viability. In CCRF-CEM T cells, TSA or azacitidine treatment resulted in increased p24 production, further indicating that TGS was responsible for the suppression. Excitingly, delivery of the aptamer construct to humanized NSG mice suppressed viremia for several weeks even after ART cessation; to this end, CD4+ T cell count also began to rebound. In contrast with previous findings, treatment did not result in significant changes to DNA methylation at the LTR. While this was initially attributed to post-transcriptional gene silencing [371], it is important to recall that the role of DNA methylation in HIV-1 latency is not fully understood, whereas histone modifications are known to be highly important. Therefore, the possibility that LTR-362 triggers heterochromatin formation in vivo cannot be ruled out.

#### 4.2.3. ASP

An open reading frame (ORF) in the complementary strand of the HIV-1 genome, located in *env*, was identified over 30 years ago in a study by Roger H. Miller [372]. The resulting 2242 bp transcript, *ASP* (short for antisense protein, sometimes called *AST* in some sources), encodes a 189-residue transmembrane protein. During latency, ASP is localized to the nucleus, but reactivation causes it to translocate to the plasma membrane; during budding, it is incorporated into the viral envelope as a structural protein [373]. Interestingly, *ASP* is also known to suppress HIV-1 gene expression and replication, and cells expressing *ASP* are resistant to latency reversal [374,375], suggesting an epigenetic mechanism of control.

In Jurkat T cells expressing *ASP* under the control of the CMV promoter, HIV-1 replication was significantly suppressed when compared to control cells. In addition, RNAPII recruitment to the 5′ LTR was reduced, even after cells were treated with TNF-α. Further experiments revealed that *ASP* recruited EZH2 to nuc-1, resulting in increased levels of H3K27 methylation. In HIV-1-infected H9 cells, which express *ASP* under control of the 3′ LTR, it was found that *ASP* physically associates with EZH2 and SUZ12—both of which are components of PRC2 [375].

Additional studies investigating HIV-1 antisense transcripts revealed a long variant of *ASP*, termed *ASP-L.* When delivered to MAGIC-5A or MOLT-4 cells, *ASP-L* significantly decreased viral replication. To this end, targeting *ASP-L* with shRNAs rescued HIV-1 gene expression. Specifically, the shRNA that targeted the downstream region of *ASP-L* was most effective at restoring gene expression [376].

A recent study from Li et al. identified the domains of the transcript that contribute to *ASP*-mediated TGS. The 5′ terminus, which corresponds to the U3 region, was required for interaction with the 5′ LTR. The downstream domain B (nt 927–1476) was responsible for recruiting PRC2, and deletion or mutation of this sequence resulted in latency reversal and a loss of histone methylation at nuc-1. Domains C and D (nt 1477–2026 and nt 2027–2574, respectively) were also implicated in the recruitment of other chromatin-modifying factors, such as HDAC2. Notably, *ASP* could potently suppress latency reversal when delivered to pCD4s isolated from PWH despite quasispecies diversity [377].

Taken all together, the results of these studies are very promising for the “Block and Lock” strategy. Engineering the *ASP* sequence to include only the necessary domains for LTR binding and factor recruitment could also potentially overcome challenges related to packaging and delivery, thus enabling effective usage of this broad-spectrum yet highly HIV-1-specific strategy.

### 4.3. Other LPAs

Tat inhibition and TGS are by far the most popular strategies to promote HIV-1 latency, due to their specificity and potency. However, as we have discussed previously in this manuscript and others (see reference [16]), proviral latency is regulated by a myriad of cellular processes. As we will discuss in this section, a number of other compounds—particularly epigenetic modifiers—have been investigated for their function as LPAs (summarized in Table 3).

#### 4.3.1. Histone Acetyltransferase Inhibitors

As we have discussed extensively in this work and others (see reference [16]), acetylation of nuc-1 by HATs, particularly p300/CBP, is one of the key steps in proviral gene expression. HAT inhibitors (HATis) are thus an attractive option in the “Block and Lock” strategy as they could maintain tight chromatin interactions to prevent access to the proviral TSS by transcriptional machinery.

Curcumin is a polyphenol derived from the rhizome of *Curcuma longa*, or turmeric. It has been mostly observed to have antioxidant and anti-inflammatory effects by acting through a number of different biological pathways (reviewed in [385]). Notably, curcumin has also been found to specifically inhibit p300/CBP by a noncompetitive mechanism without affecting the activity of other HATs such as PCAF (Table 3) [378,386]. SupT1 cells co-cultured with HIV-1-infected H9 cells had reduced syncytia formation in the presence of curcumin, indicating inhibited infection and proviral gene expression. Additionally, curcumin treatment was able to inhibit the acetylation of Tat [378], which is in agreement with the observation that p300/CBP acetylates Tat to promote its activity [387]. To this end, in HEK293T and TZM-bl cells expressing Myc-tagged Tat, curcumin treatment inhibited its ability to transactivate HIV-1 gene expression and even promoted its degradation [379]. Interestingly, the synthetic analog curcumin A has been found to have no effect on transcription after integration, but instead inhibited RT activity [388].

Garcinol is a polyisoprenylated benzophenone derived from the fruit rind of *Garcinia indica*. Unmodified, it is a nonspecific HATi and has cytotoxic effects (Table 3). p300/CBP-specific derivatives of garcinol developed by Mantelingu et al. were shown to be able to induce conformational changes in the enzyme structure and potently inhibit acetylation of H3 and H4 in HeLa cells. In the SupT1/H9 co-culture model described previously, SupT1 cells showed reduced syncytia formation and p24 production in the presence of one of the derivatives, LTK-14. Notably, histone acetylation was not fully restored in cells treated with combinations of the HATis and TSA [380], suggesting that HATis could be effective “locks” against HIV-1 reactivation.

#### 4.3.2. Histone Methyltransferase Inhibitors

Histone methylation-targeting LPAs are less well characterized. However, Nguyen et al. have recently utilized the UTX-1 inhibitor GSK-J4 to induce proviral latency (Table 3). As we have discussed previously, UTX-1 is a demethylase of H3K27 and is associated with HIV reactivation [111]. In Jurkat E4 and 3C9 cells, GSK-J4 increased H3K27 methylation levels over time, and treated cells were desensitized to TNF-α or SAHA stimulation. In agreement with these findings, GSK-J4 not only enhanced transcriptional silencing in pCD4s but also prevented proviral reactivation following TCR stimulation. Importantly, H3K27 methylation was sustained after treatment withdrawal, but these cells were re-sensitized to latency reversal stimuli [381]. UTX-1 inhibition may therefore be an effective adjuvant for long-term therapies, such as TGS.

#### 4.3.3. Bromodomain Inhibitors (LPA)

As we have discussed previously in this work, bromodomain inhibitors like JQ1 are generally associated with latency reversal because of their ability to relieve competition between Tat and P-TEFb, and displace the BAF complex from nuc-1. However, recently, Niu et al. have identified a BRD4-specific small molecule, ZL0580, that is actually an LPA (Table 3). Structure–function studies of ZL0580 compared to JQ1 showed that it is also bound in the acetyl-lysine pocket, but it additionally made contact with the WPF shelf and ZA channel [274]. J-Lat 10.6 cells treated with this compound had reduced proviral transcription and were more resistant to stimulation by prostratin or SAHA. ZL0580 alone was also able to suppress proviral gene expression in pCD4s from PWH. Interestingly, ZL0580 reduced Tat’s binding to CDK9 and induced repressive chromatin formation at the 5′ LTR [382]. However, whether the compound also displaced BAF from nuc-1 is not known.

#### 4.3.4. FACT Inhibitors

In this work, we previously discussed the role of curaxin CBL0137 as an LRA. However, another curaxin, CBL0100, was demonstrated by Jean et al. to promote HIV-1 latency rather than reactivation (Table 3). CBL0100 could reduce GFP expression in J-Lat A1 and A2 cells, as well as luciferase expression in TZM-bl cells. In pCD4s from VS-PWH, it reduced viral RNA production and showed synergistic effects when combined with antiretrovirals; interestingly, in ex vivo-infected CD4+ T cells, ART did not show this synergy. Importantly, the effect of CBL0100 was transient in these cells. CBL0100 also reduced elongated transcription in TNF-α-stimulated U1 cells, and this was associated with an expected decrease in occupancy of FACT components and RNAPII at the proviral promoter [383].

Another small molecule, Q308, was identified by Zhou et al. to suppress HIV-1 transcription (Table 3). Q308 reduced GFP expression in PMA-stimulated J-Lat 10.6 cells and likewise desensitized the cells to prostratin and SAHA. These results were corroborated in ex vivo-infected pCD4s, in which Q308 could suppress viral reactivation after TCR stimulation. Similar to what was seen with CBL0100 treatment, FACT complex occupancy was decreased at nuc-1, and further investigation revealed that Q308 facilitates proteasomal degradation of Tat and FACT proteins, but not Gag, without affecting mRNA expression. Interestingly, Q308 also induced apoptosis in ACH2 and U1 cells, but not in their uninfected parental lines [384], suggesting an additional antiviral role. Taken all together, these and previous studies suggest that FACT inhibitors may be LRAs or LPAs. Understanding the precise role of the FACT complex in HIV-1 latency will aid in selecting novel FACT inhibitors to regulate HIV-1 latency.

## 5. CRISPR/Cas

The CRISPR/Cas system (CC) is unique among the current cure strategies because it enables a level of precision and specificity unachievable by traditional LPAs/LRAs and is less invasive than stem cell replacement therapy. CC utilizes three core components: the target DNA sequence, the bacterially derived Cas endonuclease, and a 20-nucleotide guide RNA (gRNA) that is complementary to the target. On the DNA strand opposite the target sequence is an additional component, a short sequence of nucleotides known as a protospacer-adjacent motif (PAM) (reviewed in [389,390,391]). Each species of Cas recognizes a different PAM; for example, Cas9 from *Streptococcus pyogenes* (SpCas9) recognizes NGG, whereas *Staphylococcus aureus* (SaCas9) recognizes NNGRRT (reviewed in [389,391]), but Cas proteins have also been engineered to alter their PAM recognition (reviewed in [391]).

Briefly, the Cas endonuclease probes the genome of interest for PAM sites. Stable interaction between Cas and the PAM induces a conformational change that allows the Cas/gRNA complex to interrogate the protospacer region for complementarity. Successful, stable formation of the DNA-RNA hybrid activates Cas’s endonuclease activity. Normally, this results in a double-stranded break that, in eukaryotes, is typically repaired by non-homologous end-joining (NHEJ), microhomology-mediated end joining (MMEJ), or homology-directed repair (HDR) (reviewed in [390,391]). However, this endonuclease activity can be inactivated by specific mutations, resulting in a catalytically dead Cas (dCas) enzyme. Therefore, instead of cutting the DNA, dCas—often dCas9—remains bound to the target site [392]. While dCas9 itself can function as a block to transcription and replication, its N- and C-termini can be fused to other proteins that can control gene expression. In this section, we describe some CC-based strategies that have been investigated as potential HIV-1 cures.

### 5.1. CRISPR/Cas to Affect Proviral Latency

To promote proviral latency, dCas9 is typically fused to a Krüppel-associated box (KRAB) protein, which typically functions as a transcriptional repressor, partially due to its ability to promote heterochromatin formation (reviewed in [393]). dCas9-KRAB co-expressed with anti-LTR gRNAs suppressed proviral gene expression in J-Lat 6.3 and 10.6 cells, with TAR-specific gRNAs proving the most effective [394,395]. In infected HEK293T cells, this repression was sustained for up to 14 days [394]. Additionally, dCas9-KRAB blocked reactivation by PMA, SAHA, and panobinostat [394,395], indicating the formation of repressive chromatin at the provirus. Indeed, dCas9-KRAB reduced acetylation of H3 at the nuc-1 site and increased trimethylation of H3K9 [394].

In a similar vein, dCas9 fusion proteins may also be used to reverse latency. In this strategy, dCas9 is fused to a transcriptional activator, such as VP64. When complexed with gRNAs targeting U3, dCas9-VP64 induced LTR-driven luciferase expression in TZM-bl and HEK293T cells; gRNAs near the NF-κB binding site, but not overlapping it, were particularly effective. Additionally, dCas9-VP64 fused to MS2-p65-HSF1 (dCas9-SAM) was even more effective than dCas9-VP64 alone. These results were corroborated in infected Jurkat lines and CHME5 cells; however, these cells also experienced cytotoxic protein buildup and died as a result [396]. Bialek et al. also found that dCas9-SAM was highly effective at activating HIV-1 gene expression in J-Lat 6.3 and HIV-visible-transduced Jurkat T cells and could induce the production of infectious virions in J89 cells [397]. Interestingly, dCas9-SAM with a gRNA targeting the R region was highly effective in J-Lat 10.6 cells, and this was even more significant in dCas9-p300 [398], possibly due to the close proximity of p300 to nuc-1.

Taken all together, these studies suggest that dCas9 fusion proteins are highly effective as both LPAs and LRAs. They are also advantageous over small molecules, because they are long-acting and specific to HIV-1. Therefore, they would presumably have far fewer off-target effects, including neurotoxicity.

### 5.2. CRISPR/Cas Gene Editing

As we have discussed previously, ex vivo gene editing of HIV-1 coreceptors has been proposed as a cure strategy, based on the success of CCR5Δ32 transplants. However, CC-based inactivation of proviral gene expression has been investigated since at least 2013 [399]. The basic principle behind this strategy lies in the downstream effects of Cas-induced cleavage. NHEJ and MMEJ can be error-prone, leading to disruptive insertions, deletions, inversions, and substitutions (collectively, “edits”) at the target site. Therefore, Cas-induced breaks in the integrated provirus could result in edits that inactivate HIV-1 gene expression and reactivation, thus eliminating the proviral reservoir once and for all (reviewed in [27]). To this end, simultaneous breaks in the 5′ and 3′ LTR could result in complete excision of the proviral genome [400]. Our group and others have identified a number of gRNAs that are highly effective against HIV-1 and SIV in cell culture and in vivo [364,399,401,402,403,404,405,406,407,408,409]; however, none of these gRNAs has thus far been able to eliminate 100% of proviruses in all systems.

While CC-based gene editing does not necessarily target proviral chromatin, it is well known that nucleosomes impede Cas’s endonuclease activity by restricting access to the PAM site, probably as a result of steric interference or other nonspecific interactions between Cas and histone proteins [410,411,412]. Indeed, when delivered to transiently pseudoinfected HEK293T cells, anti-TAR gRNAs were nearly 100% effective in inactivating HIV-1 gene expression; however, when delivered to J-Lat 10.6 cells, their efficiency fell to about 50% [401]. Because nuc-1 is positioned over the TAR under latent conditions, it is likely that it could restrict Cas-mediated binding and cleavage, and therefore inactivation.

Notably, nuc-1 is not a stably positioned nucleosome, which allows it to be easily remodeled to facilitate processive transcription. Unstable nucleosomes undergo a process called “breathing,” whereby they dynamically and transiently wrap and unwrap. In the context of normal host gene expression, this exposes sites to TFs and other proteins [413]. Isaac et al. demonstrated that this process also exposes and hides PAM sites, thus putting Cas’s endonuclease activity at the mercy of breathing kinetics [411]. However, in the context of CC-based editing of the HIV-1 provirus, this also indicates that nuc-1 remodeling could also facilitate more favorable inactivation conditions.

Indeed, when HIV-1-infected cells were stimulated via IL-2 and TCR engagement prior to CC treatment, proviral gene expression was completely ablated in downstream assays [407]. Under latency reversal conditions such as these, nuc-1 is repositioned downstream of the TSS and additionally becomes less tightly bound to its associated DNA (Figure 1). Other studies have shown that treating cells with HDACis improves Cas-mediated cleavage of cellular genes [414,415,416]; many of these HDACis are also under investigation as LRAs. Moreover, computational analysis from our group indicates that Cas requires less DNA accessibility than RNAPII for its activity [417], indicating that total latency reversal may not be necessary to create conditions that are favorable to Cas-mediated cleavage. Taken all together, we have previously proposed the strategy “Tickle and Tweeze” (see reference [16]), whereby LRAs are used at subtherapeutic doses to open the chromatin at the 5′ LTR enough to facilitate HIV-1 cleavage (i.e., “tweeze”) without inducing proviral gene expression or viral rebound (i.e., “tickle”). While the effects of subtherapeutic doses of LRAs on proviral chromatin are not completely understood, the fact that nuc-1 remodeling takes place independent of—but is critical for—transcription initiation suggests that there may be a “sweet spot” where the LTR DNA becomes accessible without recruiting transcriptional machinery.

## 6. Challenges and Opportunities Facing the Field

HIV-1 is a lentivirus that integrates into the host genome as a provirus, where it remains “hidden” from immune surveillance while using host cell transcriptional machinery to synthesize viral mRNAs and full-length copies of its RNA genome. Whether the infection is latent or productive is regulated in large part by the same epigenetic processes that control normal host gene expression. As we have reviewed in this work, these processes have been investigated as therapeutic targets to control the activation state of the provirus, ultimately towards developing a curative strategy against HIV-1. While “Shock and Kill” and “Block and Lock” essentially have opposite goals with regard to their effects on proviral latency, they are both faced with a common challenge: targeting the diverse proviral reservoir.

HIV-1’s tropism is mostly restricted to cells expressing CD4 and CCR5/CXCR4. However, these coreceptors are expressed on a number of different cell types, including CD4+ T cells, circulating monocytes and macrophages, and tissue-resident macrophages such as Kupffer cells and microglia. HIV-1 has also been found to infect non-immune cells such as hepatocytes and astrocytes. Therefore, the reservoir consists of diverse and expansive cell and tissue types that require additional considerations for efficacy and delivery.

Indeed, a majority of investigations into LRAs and LPAs have involved T cell-derived models of latency. However, a 2013 study by Spina et al. demonstrated that these lines can still be highly diverse in their response to reactivating stimuli, including standard LRAs [418]. Even different J-Lat clones, which are derived from the same parent culture [193], have been noted to have variable sensitivities [418,419], likely due to the unique chromatin environment at each integration site. To this end, the 3D chromosomal architectures in T cells and microglia have different responses to LRAs, with microglia undergoing much more drastic chromosomal rearrangements than T cells (reviewed in [16]). However, another study showed that the mitogen concanavalin A significantly altered chromosomal positioning in Th17 cells [100], suggesting that the mechanism of reactivation may play a role.

Another important consideration in model selection is the baseline transcriptional activation state of the proviruses being studied. Many models of HIV-1 latency and gene expression are based on immortalized cell lines, which are typically actively dividing and therefore have more resources available to be exploited by HIV-1 gene expression. Ex vivo models derived from PWH are, by contrast, typically comprised of metabolically quiescent cells. Additionally, as we have discussed previously, LT-ART selects for proviruses integrated into highly heterochromatic regions, and latent proviruses are enriched in repressive histone modifications (reviewed in ref. [16]). Therefore, Immortalized lines may be more sensitive to reactivation by LRAs but more resistant to LPAs, while ex vivo models may experience the opposite. Given this, investigators should consider using multiple in vitro and ex vivo models to better recapitulate the diverse proviral reservoir.

Beyond cell type and integration site differences, proviruses themselves are tremendously diverse between and within PWH, and new quasispecies continue to arise even in PWH on LT-ART [12]. Moreover, many of these quasispecies harbor large deletions within the genic regions of the provirus but can still produce inflammatory viral proteins [420,421]. However, it is not well understood how proviral diversity affects nucleosome placement, DNA methylation, or other epigenetic factors. Our group has previously demonstrated that certain LTR variants are refractory to reactivation with TNF-α and/or Tat, likely due to altered TF binding and recruitment [422]. As we discussed previously, quasispecies diversity is particularly important for therapies that rely on nucleic acid interactions, such as TGS and CRISPR/Cas-based strategies. The RNAs that complex with the provirus must be broad-spectrum enough to target diverse quasispecies, yet it also must be specific to avoid negative off-target effects. Taken together, these studies suggest an axis among cell type, integration site, and quasispecies diversity, each exerting individual and collective influences on the efficacy of different LRAs and LPAs. An effective strategy to “Shock and Kill” or “Block and Lock” HIV-1 infection should consider these elements as new small molecules and other compounds are developed and investigated.

Importantly, the HIV-1 reservoir does not exist solely in the periphery; CNS cells can become infected as T cells and monocytes traffic across the blood–brain barrier (BBB) and release virions. As we have described previously in this work and elsewhere, HIV-1-infected CNS cells contribute to neuropathology by secreting viral proteins that induce inflammation and aberrant gene expression. Additionally, HIV-1-infected astrocytes in the brain have been noted to facilitate re-trafficking to the periphery [423,424,425], therefore replenishing and maintaining the circulating reservoir. A major contributor to this phenomenon in PWH who are otherwise virus-suppressed is the limited ability of current ART regimens to penetrate the BBB. In fact, ART drug concentrations are substantially lower in cerebrospinal fluid than in plasma [426].

How, then, can LRAs, LPAs, and CRISPR/Cas constructs be effectively delivered to the CNS? Perhaps the field should look to innovations in ART drug formulation. Recent studies have shown that nanoparticles containing current ART drugs, such as raltegravir and elvitegravir, were readily taken up in vitro and inhibited HIV-1 replication [427,428]. Importantly, when delivered via polymeric nanoparticles, elvitegravir could penetrate an in vitro BBB co-culture model [428]. Taken together, it stands to reason that the small-molecule LRAs/LPAs could also be delivered by nanoparticles. In fact, a recent study demonstrated that delivery of *tat* mRNA by lipid nanoparticles could reverse latency in multiple cell line models and pCD4s from VS-PWH. This effect also synergized with small molecule LRAs [303].

Viral vectors are also a common strategy for extended delivery of gene therapies, such as CRISPR/Cas or small RNAs. Common viral vectors include adenoviruses, adeno-associated viruses, and lentiviruses; all of which can be specifically engineered to express surface proteins and receptors to enable their transfer across the BBB, as well as specifically target cells expressing HIV-1 coreceptors (reviewed in [429,430]). Virus-like particles (VLPs) have similarly emerged to deliver nucleic acids and proteins, but can also be loaded with small molecules and other bioactive compounds (reviewed in [431]). Therefore, the use of VLPs could present a unique opportunity to deliver LRAs, LPAs, and CRISPR/Cas constructs specifically to cells within HIV-1’s tropism, which could lower the risk for off-target effects.

The end goal of the strategy must also be considered in selecting the appropriate bioactive compound. Small molecules are metabolized or otherwise degraded, which is ideal for “Shock and Kill”, which aims to be a relatively short-term regimen. However, this may not be sufficient to clear all proviruses in all tissues. By contrast, for “Block and Lock,” TGS may be more effective due to its long-term effects; otherwise, PWH may have to continue to administer small molecule LPAs, which is not necessarily advantageous over current ART regimens.

It is also possible that “Shock and Kill” and “Block and Lock” could be used in tandem, especially with tissue-specific delivery. “Shock and Kill” could target and clear the dividing infected cells in the periphery, while “Block and Lock” could protect against HAND and other HIV-1-associated neuropathology. Should such a combinatorial strategy be explored, investigators should carefully consider which bioactive compounds are appropriate to use together.

As we have described here, there has been a concerted effort over the past two decades to elucidate which factors are valid targets for an HIV-1 cure, and great strides have been made in “Shock and Kill,” “Block and Lock,” and CRISPR/Cas therapy. Future studies should investigate the effects of cell type, integration site, quasispecies diversity, and delivery mechanisms on the efficacy of LRAs and LPAs. Understanding how these variables relate to one another and to the HIV-1 reservoir will be critical to developing an effective strategy to holistically control and eradicate HIV-1 infection.

## Figures and Tables

**Table 1:** HDAC inhibitors used as LRAs.

HDACi Common Name	HDACi Class	Structure	Cell(s) Tested	Ref(s)
Trichostatin A	Pan	Hydroxamic acid 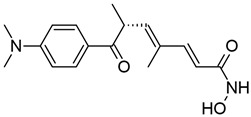	J49, OM10.1, U1	[49]
			ACH2	[49,120]
			Nuclear extract (Jurkat, HeLa, *Drosophila* S-190)	[109]
			HeLa	[119]
			HUT78-LTR-GFP	[120]
Valproic acid	Pan	Short-chain fatty acid 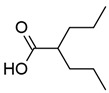	CEM-SS, Jurkat, U1	[121]
			PBMC from VS-PWH	[122,123]
			J89	[123]
SAHA	Pan	Hydroxamic acid 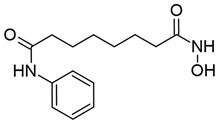	J89	[123]
			Sup-T1	[124]
			JΔK, PBMC from VS-PWH, TZM-bl	[125]
			ACH2, U1	[125,126]
			pCD4	[127]
Panobinostat	Pan	Hydroxamic acid 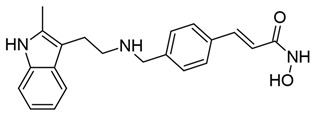	ACH2, U1	[126]
			HeLa	[128]
			PBMC from VS-PWH	[129]
Romidepsin	Class I	Bicyclic depsipeptide 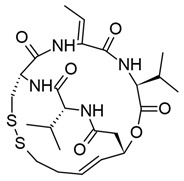	pCD4, pCD4 from VS-PWH	[130]

**Table 2:** Tat inhibitors used as LPAs.

Tat Inhibitor Common Name	Compound Type	Structure	Cell(s) Tested	Ref(s)
Nullbasic	Tat mutant	Basic domain mutant	ACH2, J-Lat 6.3	[310]
			Jurkat	[310,311]
			HeLa	[310,311,312,313]
			pCD4	[311,314]
			HEK293T	[311,313,315]
Didehydro-cortistatin A	Small molecule (synthetic)	Steroidal alkaloid 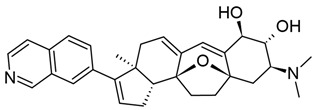	HeLa	[316,317,318,319,320]
			J-Lat 6.3/10.6	[318]
			U1	[318,319]
			OM10.1	[318,319,321]
			ACH2	[319]
			Jurkat, pCD4	[320]
			pCD4 from VS-PWH	[320,321]
			U87MG, U937	[322]
Triptolide	Small molecule (natural product)	Diterpenoid epoxide 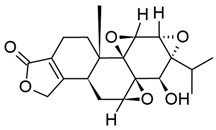	Jurkat, PBMC, TZM-bl	[323]

**Table 3:** Other compounds used as LPAs.

LPA Common Name	Compound Class	Structure	Cell(s) Tested	Ref(s)
Curcumin	HATi (p300/CBP)	Polyphenol (diarylheptanoid) 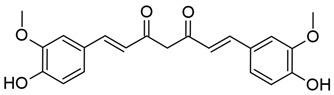	SupT1	[378]
			HEK293T, TZM-bl	[379]
Garcinol	HATi (nonspecific)	Polyisoprenylated benzophenone 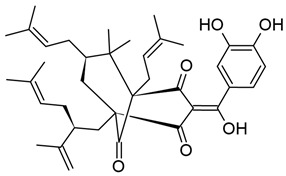	SupT1	[380]
GSK-J4	HMTi (UTX-1)	Aminopyrimidine derivative 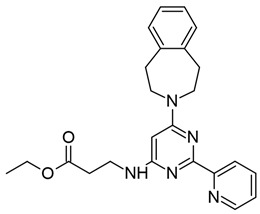	Jurkat 3C9/E4, pCD4, pCD4 from VS-PWH	[381]
ZL0580	Bromodomain inhibitor (BRD4)	Trifluoromethyl sulfonyl pyrrolidine carboxamide 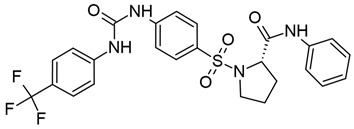	J-Lat 10.6, pCD4, pCD4 from VS-PWH	[382]
CBL0100	FACTi (curaxin)	Carbazole-like 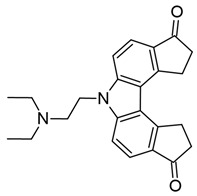	J-Lat A1/A2, Jurkat, pCD4, pCD4 from VS-PWH, THP89GFP, U1	[383]
Q308	FACTi	Quinazoline derivative 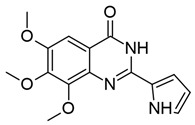	ACH2, J-Lat 10.6/A2, pCD4, TZM-bl, U1	[384]

## Data Availability

No new data were created or analyzed in this study.
